# Light-Off in Plasmon-Mediated Photocatalysis

**DOI:** 10.1021/acsnano.1c01537

**Published:** 2021-06-22

**Authors:** Christopher Tiburski, Astrid Boje, Sara Nilsson, Zafer Say, Joachim Fritzsche, Henrik Ström, Anders Hellman, Christoph Langhammer

**Affiliations:** † Department of Physics, Chalmers University of Technology, 412 96 Göteborg, Sweden; ‡ Department of Mechanics and Maritime Sciences, Chalmers University of Technology, 412 96 Göteborg, Sweden

**Keywords:** nanoalloys, heterogeneous catalysis, plasmonics, photothermal, CO oxidation, gold−palladium, photocatalysis

## Abstract

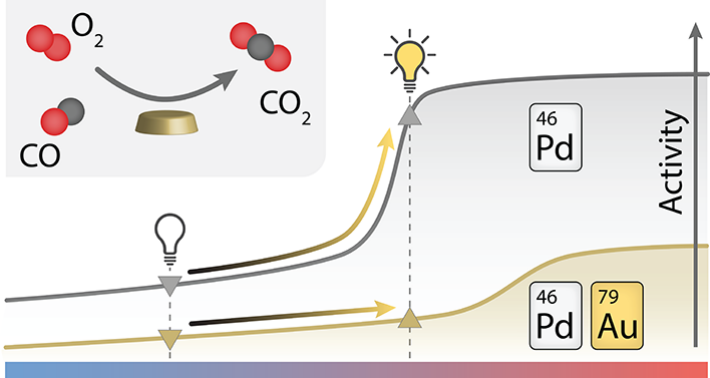

In plasmon-mediated photocatalysis it is of critical importance to differentiate light-induced catalytic reaction rate enhancement channels, which include near-field effects, direct hot carrier injection, and photothermal catalyst heating. In particular, the discrimination of photothermal and hot electron channels is experimentally challenging, and their role is under keen debate. Here we demonstrate using the example of CO oxidation over nanofabricated neat Pd and Au_50_Pd_50_ alloy catalysts, how photothermal rate enhancement differs by up to 3 orders of magnitude for the same photon flux, and how this effect is controlled solely by the position of catalyst operation along the light-off curve measured in the dark. This highlights that small fluctuations in reactor temperature or temperature gradients across a sample may dramatically impact global and local photothermal rate enhancement, respectively, and thus control both the balance between different rate enhancement mechanisms and the way strategies to efficiently distinguish between them should be devised.

Plasmon-mediated photocatalysis has seen rapid development since the seminal publications more than a decade ago.^1−9^ It builds on the excitation of the localized surface plasmon resonance (LSPR) in metal nanoparticles and the fact that, in this way, optical cross sections many times larger than the particle size can be achieved.^10^ Mechanistically, the LSPR phenomenon may influence a chemical process on a metal nanoparticle surface in three distinctly different ways, which, in principle, may manifest themselves individually or in concert: (i) photothermal heat generation, (ii) optical near-field enhancement, and (iii) direct hot-charge carrier generation in the metal and their injection into surface-adsorbed reactant species.^11−13^ Ever since the hot-carrier mechanism was first proposed, one of the most important and critical questions has been how to distinguish it from the photothermal one. As a consequence, being able to appropriately answer this question has developed into a key effort in the field, spurred significantly by a recent controversy on data interpretation in key studies,^13−18^ and discussions of experimental procedures for distinguishing photothermal from hot-carrier reaction enhancement processes.^19,20^

Focusing on the photothermal mechanism, we remind ourselves that upon decay of an LSPR in a metal nanoparticle, the excited electrons will relax via electron–phonon coupling within picoseconds, unless steered otherwise.^21,22^ In other words, the electronic excitation is transferred into heat via coupling to the phonon bath of the nanoparticle, which further dissipates the heat to the nanoparticle surrounding and support, and reaches thermal equilibrium on the 10–100 ps time scale.^18,21^ As a consequence, temperature gradients may form due to (i) nonuniform metal nanoparticle distributions, (ii) inhomogeneous illumination intensity, (iii) 3D nano- or mesoporous support materials with complex and anisotropic heat transport properties, and (iv) attenuation/absorption of irradiated photons inside such a support material. These effects have been identified as important and pinpointed as inadequately addressed in the literature.^14,18^

A further important factor in this context is the fact that the reaction rate of a thermally activated catalytic reaction does not depend linearly on temperature. In the kinetic regime, when the rate is relatively low, its temperature dependence can be described by the Arrhenius equation, which states that the rate constant is defined by *k* = *A* exp(−*E*_a_/RT), where *A* is the pre-exponential factor, *E*_a_ the molar activation energy, *R* the gas constant, and *T* temperature. As a first key aspect, this means that the reaction rate increases exponentially with temperature. Consequently, in a photothermal process, provided the reaction is taking place in the kinetically limited regime, the rate is expected to exhibit an exponential dependence on the illumination power.^20^ Therefore, the most widespread approach to distinguish between photothermal and direct hot-carrier mediated reaction mechanisms has been to study a reaction at increasing photon flux and investigate whether this dependence is linear or exponential—an approach that has been identified as problematic and to yield ambiguous results.^20^ The main reason for this ambiguity is that in corresponding studies only limited ranges of photon flux variation have been used since actually implementing the required several orders of magnitude difference in photon flux would require the use of very high-power light sources and/or the use of ultrasensitive detection techniques at very low reaction rates.

As a second key aspect, we note that in a real system the increase of the reaction rate cannot continue to infinity, since, at a certain point, diffusion limitations and mass transport gradients will start to dominate the process. This leads to a slowing down, and eventually a complete halt, of the rate increase, once the system is operated entirely in the mass transport limited regime. In thermal catalysis, this behavior is captured in a so-called light-off curve, which typically is a plot of the reaction rate (or conversion) as a function of temperature. As the key implication here, the photothermal enhancement will be strongly dependent on *where* on the light-off curve the catalyst finds itself during the experiment. In other words, the same photon flux can lead to rate enhancements that differ by several orders of magnitude, depending on the starting temperature of the catalyst. Consequently, for example, small fluctuations in reactor temperature, as well as temperature gradients across a sample, may have a dramatic impact on the (local) photothermal rate enhancement and the balance between the three potential plasmonic rate enhancement mechanisms when they take place in concert. Surprisingly, however, this aspect is rarely addressed in the debate on enhancement mechanisms in plasmon-mediated catalysis and the corresponding discussion of experimental strategies to efficiently distinguish between them.

In response, we report here a study that explicitly demonstrates the importance and potential magnitude of this effect on the example of carbon monoxide (CO) oxidation over two-dimensional (2D) nanofabricated Pd and Pd_50_Au_50_ alloy model catalysts. We have chosen these specific systems because they by design effectively eliminate 3D temperature gradients within the catalyst material due to their 2D nature and because alloying Pd with Au constitutes an efficient way to alter the light-off behavior of the reaction,^23,24^ while, as we demonstrate, essentially not altering light absorption, and thus the photothermal properties of the system.

## Results and Discussion

### Sample Characterization

**Figure 1:**
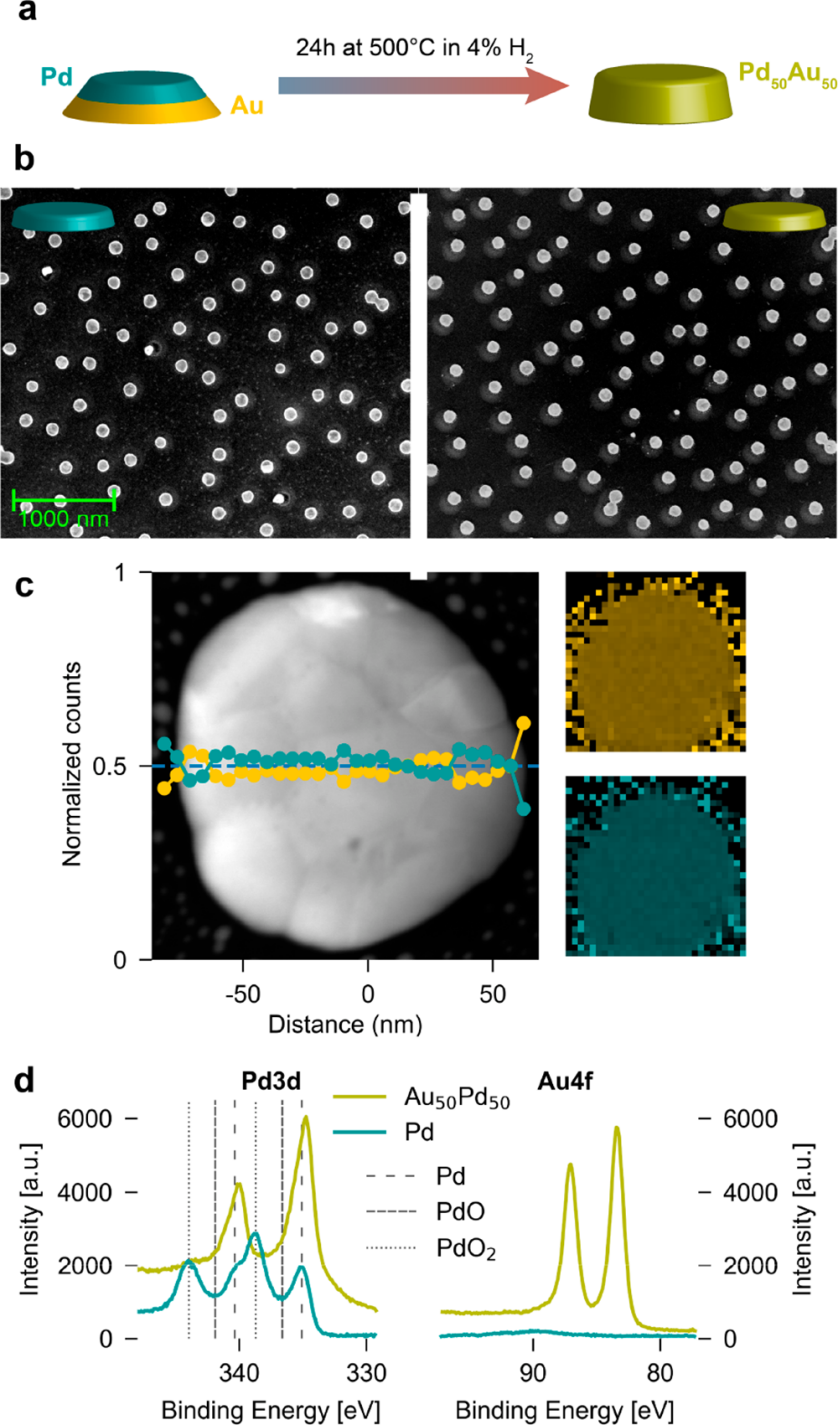
Pd and Au_50_Pd_50_ sample characterization. (a) The alloy nanoparticle array is manufactured by subsequently evaporating a thin Au and Pd film through a nanofabricated mask, with thicknesses tailored to yield the targeted alloy composition. Subsequently, alloy formation is induced by annealing the sample at 500 °C for 24h in 4% H_2_ in Ar carrier gas.^26^ (b) SEM images of annealed neat Pd (left) and Au_50_Pd_50_ alloy (right) nanodisk arrays. (c) HAADF-STEM image of an Au_50_Pd_50_ alloy nanodisk, depicted together with both an elemental line scan and a TEM-EDS map of the Pd and Au constituents. The line scan confirms the homogeneous distribution of both elements across the particle. (d) XPS spectra of a neat Pd and an Au_50_Pd_50_ alloy nanoparticle array taken before catalysis experiments for the Pd 3d (left) and Au 4f (right) peak regions, revealing a surface composition of 46 at % Au and 54 at % Pd for the alloy, as well as some degree of PdO and PdO_2_ formation (Figures S1 and S2) corroborated also by SEM imaging (Figure S3).

For our study, we have nanofabricated large-area arrays of Pd and Pd_50_Au_50_ alloy nanoparticles by hole-mask colloidal lithography^25,26^ onto 18 mm × 9 mm fused silica substrates (Figure 1a and b). The resulting Pd and Pd_50_Au_50_ nanoparticles have a disk shape with an average diameter of 113 and 132 nm and a height of 38 and 28 nm, respectively (see Methods for details). Transmission electron microscopy–energy dispersive X-ray analysis (TEM-EDX) reveals homogeneous and uniform alloy formation across the Pd_50_Au_50_ particles (Figure 1c).^26^ X-ray photoelectron spectroscopy (XPS) furthermore reveals a surface composition of 46 at % Au and 54 at % Pd, which is in good agreement with the targeted value, and due to the surface sensitivity of XPS, again also confirms homogeneous alloy formation (Figure 1d).

### CO Oxidation in the Dark

**Figure 2:**
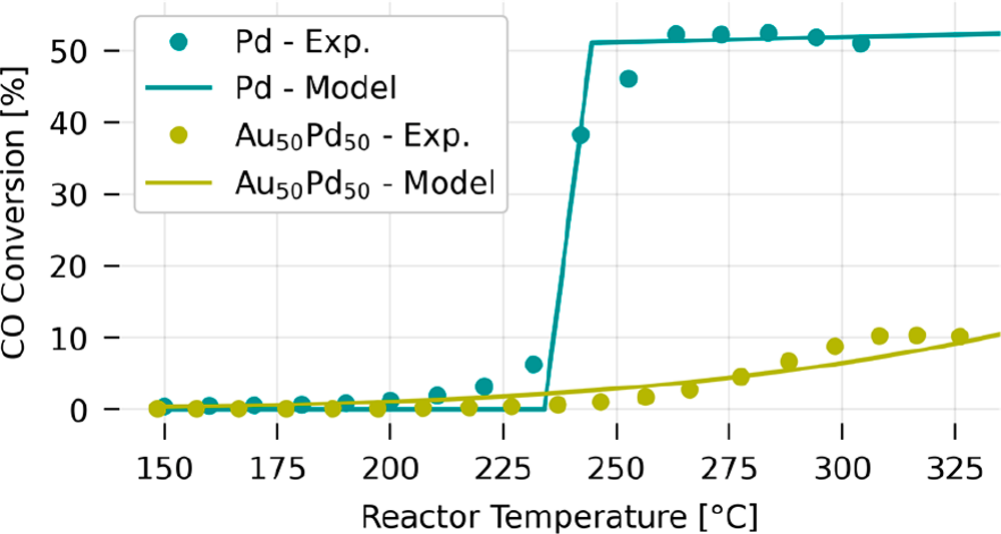
Pd and Au_50_Pd_50_ light-off curves in the dark. The light-off curves for an array of neat Pd and Au_50_Pd_50_ alloy nanoparticles were obtained using a gas flow rate of 200 mL/min in a gas mixture of O_2_ and CO in Ar carrier at a constant relative CO concentration α_CO_ = 0.2 and a total reactant concentration of 10%, by increasing sample temperature in the dark in ∼10 °C steps from ∼150 to ∼325 °C (see also Figure S5). The solid lines show the conversion predictions for a CSTR model (see the Kinetics section of the SI) using the same conditions as in the experiment (Table S4), i.e., a residence time of 4.5 s and inlet pressure of 1 atm. The Pd catalyst model was a Pd(111) surface and the Au_50_Pd_50_ model was a mixed PdAu surface with an Au top layer (see Tables S1 and S2). The number of active sites in each model system was chosen to match the maximum experimentally observed conversion since the exact number of sites in the experiment is unknown.

For the catalysis experiments, we have utilized an externally heated quartz-tube plug-flow-type reactor with an integrated “glass pocket”, as reported by Fredriksson et al.^27,28^ This pocket minimizes dilution of reaction products and thereby enables quadrupole mass spectrometric (QMS) analysis of reaction products from our nanofabricated 2D samples (Figure S4). The CO oxidation experiments were conducted with a total gas flow rate of 200 mL/min, which introduces a flow rate of 2.4 mL/min through the pocket, in a gas mixture of O_2_ and CO in Ar carrier at a constant relative CO concentration  = 0.2 and a total reactant concentration of 10%. During the experiment, the sample temperature in the dark (i.e., without illumination) was increased in ∼10 °C steps from ∼150 to ∼325 °C (Figure S5). Temperature control was implemented by setting a constant power output of the heating system, that is, *without* using an active feedback loop. This is important since an active feedback system would reduce the heating power supplied during illumination to compensate for the light-induced heating of the sample. To ensure a constant temperature at each temperature step, we instead equilibrated the system for 150 min (Figure S5). By then extracting the CO conversion at the end of each temperature step, we constructed the light-off curves for neat Pd and the Au_50_Pd_50_ alloy system in the dark (Figure 2). In the low temperature regime, where the reaction is slow enough not to be limited by mass transport, the reaction rate is kinetically limited. Upon further temperature increase, the system reaches and passes through a transient regime, where a transition from kinetically controlled to mass transport-controlled conditions takes place. This yields the typical S-like light-off curve for both systems. However, as the key result we highlight that the transition to mass-transport control occurs at lower temperature for the neat Pd catalyst compared to the Pd_50_Au_50_ system and that conversion for the latter is generally significantly reduced.

In a first analysis step to rationalize the differing light-off behavior observed between Pd and Au_50_Pd_50_ in Figure 2, we conducted first-principles informed microkinetic modeling. Specifically, since the exact surface structure of the alloy used in the experiment is unknown and the surface may undergo rearrangement in the presence of adsorbates, we studied three simplified configurations with DFT (details in the first-principles and kinetics calculations section of the SI and structures in Tables S1 and S2) and found a configuration with an Au top layer to be most thermodynamically favorable, followed by a perfectly mixed Pd–Au surface. However, in reality a blend of configurations is likely to coexist on the alloy catalyst, with local reactivity depending on the presence of Au and Pd atoms at the surface. Next, we calculated the adsorption energies of CO and oxygen on the three chosen surface configurations and found reduced binding energies for the systems with Au atoms in the top layer (Table S3). Then, we predicted the transition state energies for CO oxidation using the scaling relation derived by Falsig et al.,^29^ indicating a reduced activation energy with increasing presence of Au atoms on the surface (Table S3).These weaker binding energies and reduced barriers with increasing Au fraction are consistent with previous first-principles calculations of the PdAu alloy system.^30^

### First-Principles Informed Microkinetic Modeling

To now investigate the impact of these different energetics on the expected light-off behavior of the catalyst, we modeled the pocket reactor used in the experiments as a continuously stirred tank reactor (CSTR) with residence time and conditions chosen to match the experimental setup (details in the Kinetics section of the SI and Table S4). Specifically, we tested the energetics obtained for each modeled surface configuration and estimated the unknown exact number of active sites in the experiment to match the experimentally observed maximal conversion for each system. The correspondingly obtained results for the neat Pd surface reproduce the measured conversion profile very well (Figure 2). For the alloy system, we obtained the best agreement with the experiment for the most thermodynamically favorable model surface (i.e., the surface with an Au top layer), which showed reduced surface concentrations of reactants and correspondingly lower conversion. Smoother conversion profiles, lacking a sharp light-off point, were obtained for the model systems with Au atoms in the top surface layer due to the reduced kinetic barriers in these systems. This suggests that both reduced binding energies and reaction barriers for the alloy system, consistent with gold atoms near the surface, explain the differences in the magnitude and shape of the conversion profiles observed for the neat Pd and Au_50_Pd_50_ systems. Taken together, the first-principles calculations and kinetic modeling results thus provide a qualitative rationalization for the observed conversion profiles based on the respective energetics. However, we also highlight that they do not explicitly describe the likely dynamic surface restructuring taking place in the real catalyst (to do so a more complex model would be required, which is beyond the scope of this work) and thus do not mean that a continuous Au top layer is formed. Rather, they should be interpreted as that an Au-rich surface is formed and the reason for the experimentally observed differences between the pure Pd and the Au_50_Pd_50_ light-off curves.

### CO Oxidation under Illumination

**Figure 3:**
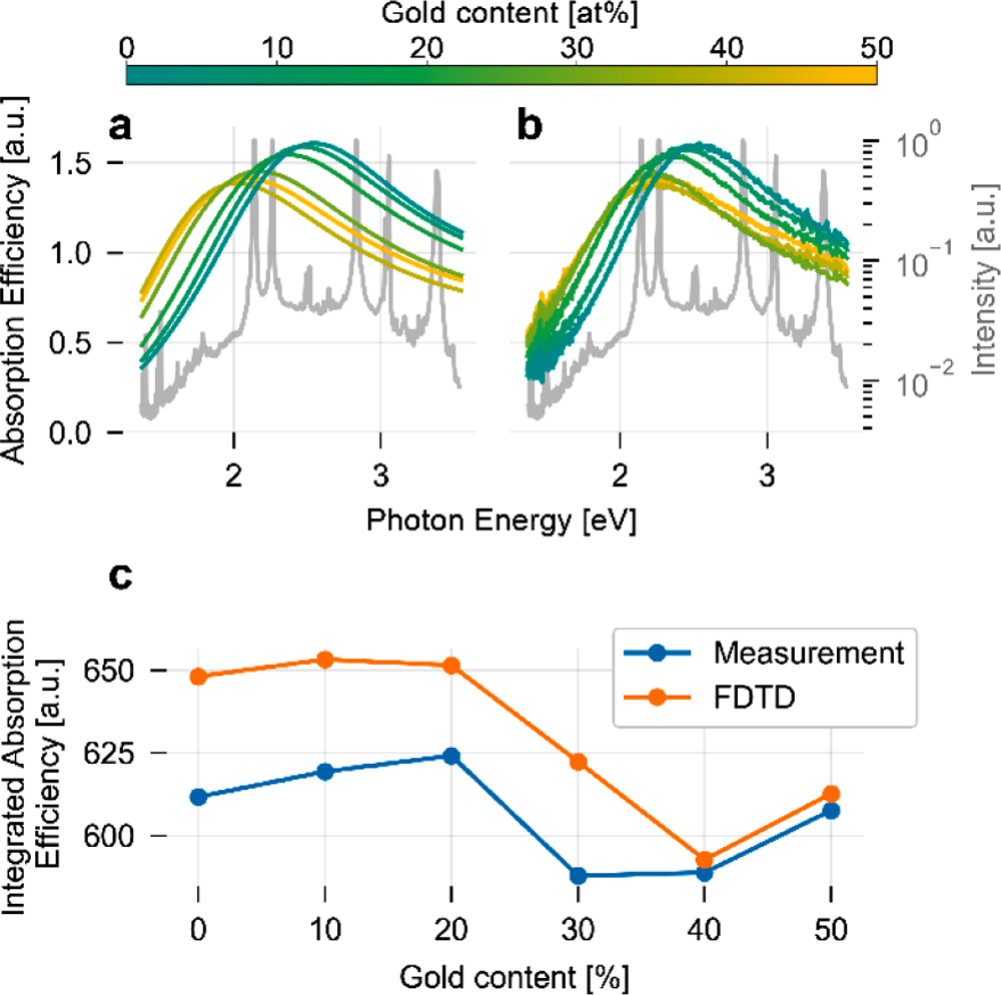
Light absorption efficiency in Pd and AuPd alloy nanodisks. (a) Absorption efficiency (absorption cross-section/nanodisk projected area) spectra for a nanodisk comprised of neat Pd and a Au_*x*_Pd_100–*x*_ alloy with its composition varied in *x* = 10 at % steps up to a composition of Au_50_Pd_50_. The spectra were obtained by FDTD simulations for a single nanodisk located on a fused silica substrate and by using the dielectric functions from the work of Rahm et al. as input.^31^ The gray line plotted in the background depicts the emission spectrum of the light source used for the photocatalysis experiments described below and confirms excellent overlap with the absorption spectra. (b) Corresponding experimentally measured absorption efficiency spectra for quasi-random arrays of neat Pd and PdAu alloy nanodisks with composition varied in 10 at % steps up to a composition of Au_50_Pd_50_. The experimental data are scaled to the FDTD data (for unscaled spectra, see Figure S6). (c) Integrated absorption efficiencies calculated from the spectra depicted in a and b for the spectral range from 1.37 to 3.55 eV. The total absorbed energy varies only by around 12%, which very likely results in a similar photon-induced temperature increase of the nanostructures upon illumination, irrespective of their composition.

Having established the CO oxidation reaction on our two catalyst model systems in the dark, we now assess their interaction with visible light. Specifically, we carried out finite-difference time-domain (FDTD) simulations of the absorption efficiency of a Au_*x*_Pd_1–*x*_ alloy nanodisk with its composition, *x*, varied in steps of 10 at % from neat Pd to Au_50_Pd_50_, using dielectric functions calculated from first-principles as the input (Figure 3a).^31^ In good agreement with our previous results assessing extinction efficiencies,^31^ increasing the Au content in the alloy shifts the LSPR absorption peak to lower photon energies. Furthermore, and as the key point here, the overall appearance of the peak, i.e., the full-width-at-half-maximum that is proportional to the LSPR dephasing time,^21,22^ remains very similar throughout the entire composition range. Nanofabricating arrays of Pd and Au_*x*_Pd_1–*x*_ alloy nanoparticles across the same compositional range, and experimentally measuring their absorption efficiencies (defined as absorption cross section/nanodisk projected area derived from SEM image analysis) using an integrating sphere detector,^10,32^ reveals good agreement with the simulations (Figure 3b) and further corroborates the similar total absorption efficiencies of neat Pd and its alloys with Au up to 50 at %. This last point becomes even more clear when plotting the integrated absorption efficiencies for Pd and its alloys as obtained both from the FDTD simulations and the experimental data (Figure 3c). Specifically, this analysis shows that the overall light absorption of an array of Pd and Au_50_Pd_50_ alloy nanoparticles is similar and, therefore, is expected to yield a similar photothermal heating effect due to light absorption, while at the same time having significantly different catalytic properties in the dark.

**Figure 4:**
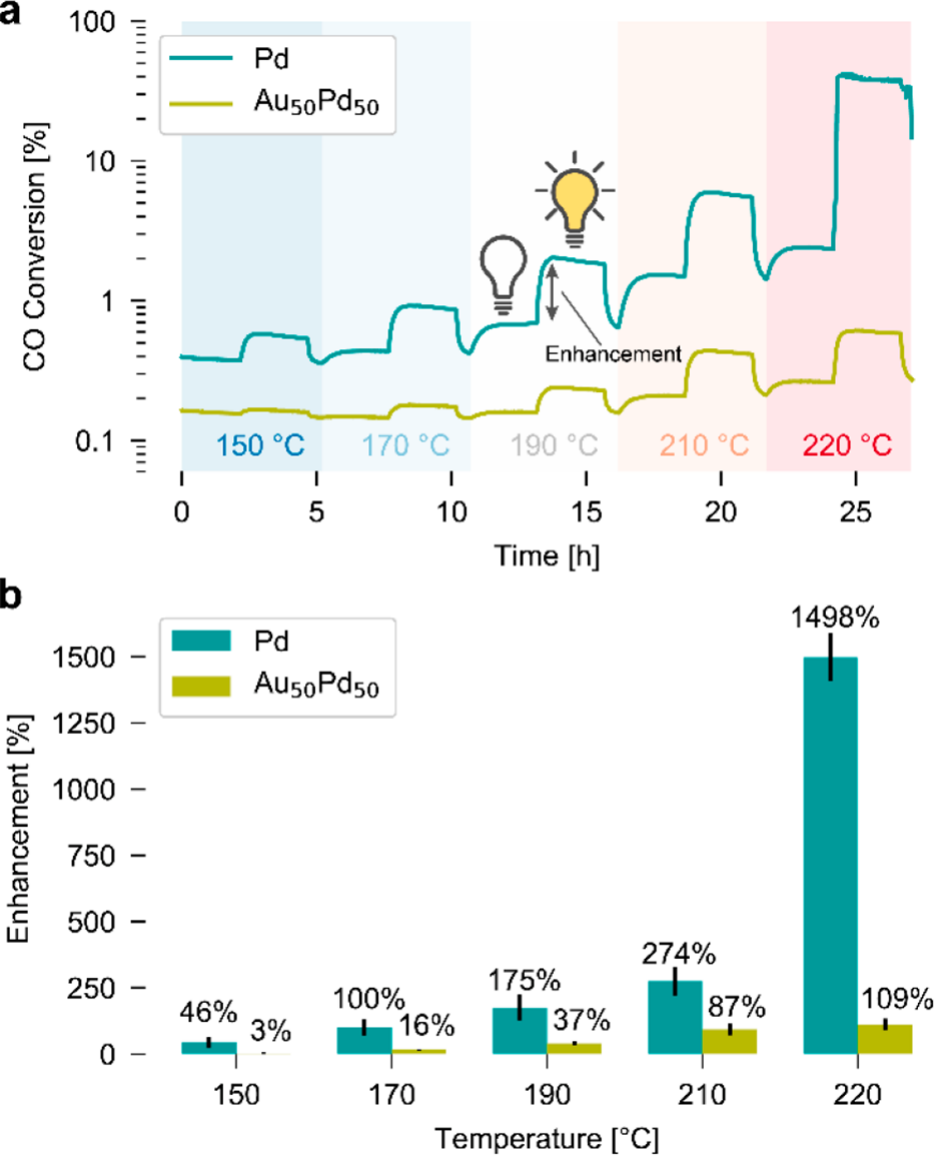
Light-induced reaction rate enhancement for constant irradiance at different catalyst temperatures. (a) The CO conversion in the dark and upon illumination for neat Pd (blue) and Au_50_Pd_50_ (yellow) measured in a constant flow of CO and O_2_ in Ar carrier gas with a relative CO concentration, , of 0.2 and at five different dark temperatures ranging from ∼150 to ∼220 °C. (b) The enhancement of the reaction rate due to constant-irradiance illumination of the two model catalysts at five different temperatures in the dark. The black lines constitute error bars that depict the standard deviation from three repetitions. Note the strong dependence of the rate enhancement on catalyst dark temperature, and the significant difference of this effect between neat Pd and the Au_50_Pd_50_ alloy system.

Turning now to assessing the impact of visible light illumination on our two model catalyst systems during reaction, we carried out an experiment where we measured the reaction rate of the two systems in the dark and under illumination of a mercury xenon arc light source at constant output irradiance of 6.8 W/cm^2^ for five selected temperatures (150, 170, 190, 210, 220 °C) along the light-off curve (cf. Figure 2). As the main result, we find a clear rate enhancement under illumination for both catalyst systems, which strongly depends on the catalyst temperature in the dark, with the dependence being distinctly nonlinear and significantly lower for the Au_50_Pd_50_ alloy (Figure 4a). Further quantification of the enhancement effect, defined as , reveals that for neat Pd at 220 °C the reaction rate is enhanced by ∼1500% upon illumination, whereas the enhancement is only ∼50% at 150 °C (Figure 4b). For the Au_50_Pd_50_ system the overall trend is similar, however, at 150 °C the light-induced rate enhancement is only 3% and at 220 °C it is 110%, which is more than a factor 10 lower compared to neat Pd.

It is now interesting to discuss these findings from a number of different perspectives. First of all, from a purely plasmonics point of view, this result may appear surprising because it is generally well-established that Au is a “better” plasmonic metal than Pd.^22,33^ Hence, one could intuitively expect that adding Au to Pd would boost the plasmon-mediated catalytic properties.^34^ Interestingly, however, we observe the opposite. Second, as we have shown (cf. Figure 3), the integrated absorption efficiency of the Pd and Au_50_Pd_50_ systems is very similar, which implies that any (photothermal) enhancement effects would be similar as well. Clearly, also this scenario is in stark contrast to our experimental results.

**Figure 5:**
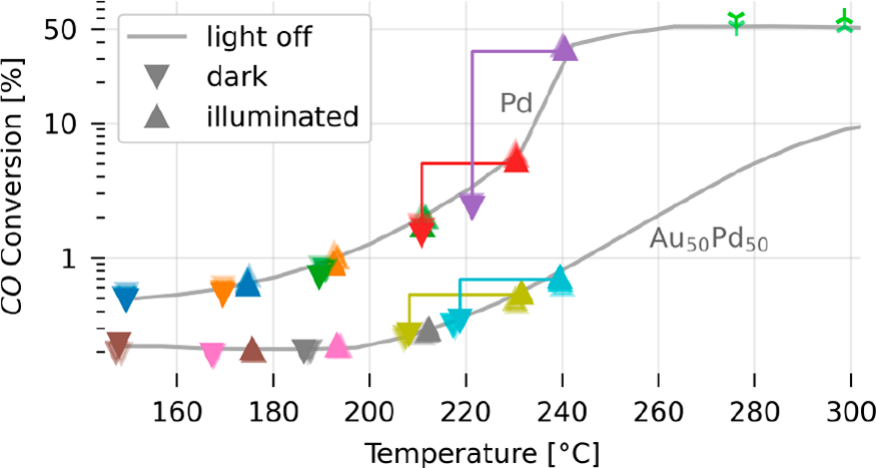
CO conversion in the dark and under illumination. CO conversion for five temperature steps under illumination (▲) and in the dark (▼) plotted versus temperature and in the same graph as the light-off curve previously measured in the dark (calibration—cf. Figure 2). The semitransparent markers represent repeated measurements. The colored lines indicate the shift of the catalyst along the light-off curve upon illumination. Also shown are two data points obtained in the full mass transport limited regime in a separate measurement under illumination (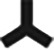) and in the dark (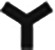), which reveal that in that limit no light-induced conversion enhancement is observed, as expected (see Figure S9 for raw data).

To nevertheless understand our findings, we first remind ourselves of the light-off curves measured for both model catalysts in the dark. They revealed a significantly lower thermal rate for the Au_50_Pd_50_ alloy compared to neat Pd for constant temperature (cf. Figure 2). Second, we extract the CO conversion for each of the five reactor temperatures in the dark and under illumination (Figure S7), together with the corresponding illumination-induced sample temperature increase measured with a thermocouple that touches the side of the sample (Figure S4b), which are on the order of 25 °C (Figure S8). Plotting the correspondingly obtained CO conversion for each temperature step under illumination (▲) and in the dark (▼) versus temperature, and in the same graph as the light-off curve previously measured in the dark (cf. Figure 2), we make the following key observations (Figure 5): (i) light-induced rate enhancement is strongly dependent on the reactor temperature; (ii) the light-induced enhancement is significantly smaller for the Au_50_Pd_50_ catalyst compared to neat Pd, irrespective of reactor temperature; (iii) the CO conversion values measured upon illumination align almost perfectly with CO conversion values of the light-off curve measured in the dark for both catalysts, irrespective of the reactor temperature; (iv) at high temperature, in the fully mass-transport limited regime, we do not observe any light-induced rate enhancement. Altogether, this clearly shows that it is the position along the light-off curve, determined by the set reactor temperature, which dictates the observed photothermal reaction rate enhancement. Furthermore, it demonstrates that the apparent significantly lower photoactivity of the Au_50_Pd_50_ alloy is solely the consequence of its intrinsically lower catalytic activity in the dark at the present conditions. These observations are further corroborated by the fact that we actually observe a slightly *reduced* light-induced sample temperature increase for higher reactor temperatures from the thermocouple reading for both samples (from ∼26 to ∼20 °C—Figure S8), while we see that the light-induced reaction rate enhancement is increased. As a final remark, we also note that the thermal equilibration between the catalyst nanoparticles and the support is very efficient even for the used fused silica support and that, therefore, thermal gradients between the particles and the support can be neglected.^35^

## Conclusions

In conclusion, we have shown that the photothermal reaction rate enhancement on nanofabricated Pd and Au_50_Pd_50_ alloy model catalysts during the catalytic oxidation of CO strongly depends on the catalyst temperature in the dark, and is solely mediated by its light-off curve. Specifically, we have found that the photothermal rate enhancement for the Pd system varies over 3 orders of magnitude at constant irradiance, depending on the position of the catalyst on the light-off curve. Simultaneously, we have identified that photothermal rate enhancements for the Au_50_Pd_50_ alloy system are significantly lower, despite very similar integrated optical absorption efficiency and thus light-induced temperature increase. As the reason we identified the overall lower catalytic activity of the alloy system compared to neat Pd, and the consequently significantly different light-off curve of the reaction in the dark. To explain this different catalytic behavior, we conducted first-principles informed microkinetic modeling showing that the lower activity of Au_50_Pd_50_ is caused by reduced binding energies of CO and oxygen, which leads to low surface coverages of both reactants, hindering the kinetic activity despite a corresponding reduction in the activation energy.

As the key implications of the above results, we highlight that understanding the light-off curve of a catalyst is critical when assessing any plasmon-mediated photocatalysis effects. Thus, as explicitly demonstrated here, a catalyst’s sensitivity toward a photothermal rate enhancement mechanism may vary by multiple orders of magnitude at constant irradiance, depending solely on the systems position along the light-off curve. Consequently, when more realistic three-dimensional catalyst systems are considered, locally, the catalyst may be operated at different positions along a light-off curve due to (i) temperature gradients across the material, (ii) attenuation of the photon flux within the sample, or (iii) locally varying mass transport limitations within a porous support. This, in turn, means that locally the balance between reaction enhancement mechanisms may be very different, further complicating discrimination between them. Finally, our results also highlight the importance of light-off characterization in plasmon-mediated catalysis when designing experimental strategies for discriminating rate enhancement mechanisms since the photothermal channel can be dramatically suppressed or enhanced, depending on the position along the light-off curve. Therefore, it should be carefully selected and optimized for each specific catalyst and reaction system.

## Methods

### Nanofabrication

All samples were fabricated using hole–mask colloidal lithography^36,37^ on either fused silica for catalysis experiments and spectrophotometry, on oxidized silicon for SEM imaging, or on TEM windows^38^ for TEM-EDS measurements. For the nanofabrication steps the following instrumentation was used: spin-coating (Suss, LabSpin6), oxygen ion etching (Plasmatherm, BatchTop m/95), electron beam evaporation (Lesker, PVD 225). To create the hole-mask, we used a 0.2 wt % a 140 nm sulfate latex bead solution (molecular probes by life technologies) in deionized water. The nanodisks were annealed in a flow reactor for 24 h at 500 °C in (4 ± 0.12) % H_2_ in Ar carrier gas at a flow rate of 200 mL/min to induce alloy formation. The annealing process leads further to a slight change in dimensions (Figure S10).

### Plug-Flow Reactor with “Pocket”

The (photo)catalysis experiments were carried out in a plug-flow type reactor (X1, Insplorion AB, Göteborg, Sweden) equipped with a custom-made pocket reactor as reported by Fredriksson et al. (Figure S4).^27,28^ Gas composition in and flow rate through the reactor is controlled by mass flow controllers (Bronkhorst Low-ΔP). The reactor is heated by a resistive heating coil and connected to a quadrupole mass spectrometer (Pfeiffer, GSD 320). Two type-K thermocouples are inserted in the reactor to monitor (i) the temperature of the gas upstream of the pocket and (ii) the actual temperature of the sample. The latter thermocouple is spring-loaded to ensure good contact with the side of the sample inside the pocket. The thermocouple is placed in the light path and therefore experiences a constant temperature increase upon illumination, which, however, is on the order of 1 °C or less (Figure S11) and the same for all experiments and catalysts. Furthermore, this heating of the thermocouple does not impact the temperature control of the reactor since the temperature reading is not used in a feed-back loop manner. The reactor has an optical port perpendicular to the flow direction to enable illumination by a 1000 W mercury xenon arc light source (Newport 6295NS in a 66921 housing), which is equipped with a liquid optical IR filter (Newport 6123NS).

Before the photocatalysis experiments, the samples were activated by a treatment of 60 min 6% O_2_, followed by 30 min 3% H_2_, and finally 60 min 6% O_2_ in Ar carrier gas with a constant flow of 200 mL/min at 500 °C. The activation was followed by a cooling period of 5h to 150 °C in Ar. Then, 1.5 h prior to the first temperature step or temperature ramp, CO and O_2_ were introduced simultaneously.

The used gases were CO (10% (±2 rel %) in Ar), O_2_ (99.9992% purity), H_2_ (4% (±2 rel %) in Ar), and Ar (99.9999% purity) as the carrier gas.

### FDTD Simulations

Lumericals FDTD solution version 8.21.2088 was utilized to calculate the optical absorption spectra presented in Figure 3. A single disk was placed on a SiO_2_ support with a refractive index of 1.46.^39^ The dielectric function for Pd and the Au_*x*_Pd_100–*x*_ alloys with *x* = 10, 12, 30, 40, 50 were taken from Rahm et al.^31^ The disk diameter (Figure S10) used in the simulations was derived from SEM images by analyzing at least 350 particles from different sample positions and averaging their individual diameters. The particle itself was modeled as a tapered cylinder (angle of 5°) with rounded edges (4 nm bottom and 6 nm top rounding). The used disk height was calculated by assuming the conservation of volume from the unannealed to the annealed particle (Figure S10). The linearly polarized plane wave light source was introduced via a total-field/scattered field source.

### X-ray Photoelectron Spectroscopy

XPS measurements were performed with a PHI 5000 (Physical Electronics). The photoexcitation was done with a monochromatized Kα-line of an Al source operated at 50 W. The energy step width was 0.1 eV, and the pass energy was 55 eV. The base pressure was always lower than 2.0 × 10^–6^ Pa. All spectra were corrected by setting the adventitious C-1s peak of the C–C bond to 284.8 eV. To ensure that this correction is physically meaningful, the Fermi level alignment was checked as suggested by Greczynski and Hultman.^40^ For quantitative analysis relative sensitivity factors from PHI MultiPak’s database for Au and Pd of 417.021 and 341.244, respectively, were employed.

### Spectrophotometry

The optical absorption spectra were obtained with a Cary 5000 spectrophotometer (Varian) equipped with a DRA2500 integrating sphere accessory. The spectra were recorded from 250 to 2500 nm with a step width of 1 nm.

### Scanning Electron Microscopy

A Zeiss Supra 55 was used to record the SEM images with the in-lens system and an acceleration voltage of 15 kV at a working distance of at least 5 mm.

### Transmission Electron Microscopy

For the STEM images and the elemental EDS maps a FEI Titan 80–300 equipped with an INCA X-sight detector (Oxford Instruments) was used. It was operated at 300 kV and lateral resolution was 5 nm for which the spectra were acquired. The acquisition time was 5 s for each spectrum, and the sample holder was tilted about 20° toward the detector to increase X-ray signal. The background was corrected for and the peaks fitted standard-less using the FEI TIA software version 4.3.

### First-Principles Calculations

Periodic density functional theory calculations were used to find relaxed geometries and energies for Pd(111) and three PdAu alloy surfaces. Calculations were performed on 2 × 2 × 3 unit cells using VASP,^41,42^ with the revised PBE^43^ generalized gradient approximation and projector augmented wave method.^44^ An energy cutoff of 450 eV was used for the plane wave basis set. The three PdAu surfaces comprised a perfect mix of Pd and Au atoms on the lower layers with (i) a perfectly mixed top layer, (ii) an Au top layer and Pd second layer, and (iii) a Pd top layer and Au second layer. Binding energies were calculated for CO and O on each surface and the gas molecules were relaxed in a 10 × 10 × 10 Å^3^ box. More information is given in the first-principles and kinetics calculations section of the SI, with structures in Tables S1 and S2, and energies in Figure S13 and Table S3.

### Microkinetic Model

Modeled surface kinetics included CO adsorption, O_2_ dissociative adsorption, and irreversible CO oxidation to form CO_2_. CO_2_ desorption was assumed to be instantaneous and irreversible. CO and O_2_ desorption were assumed to be in thermodynamic equilibrium with the respective adsorption steps. The binding energies from the DFT calculations were used as the reaction energies for adsorption and to predict the activation energies for CO oxidation on each surface using the scaling relation developed by Falsig and co-workers^29^ (Table S3). CO oxidation was modeled as a Langmuir–Hinshelwood reaction, with a prefactor from transition state theory. The free energies were computed in the harmonic limit for adsorbates and the ideal gas approximation for gases. The pocket reactor was modeled as a continuously stirred tank reactor with surface-based kinetics. The system of differential equations describing the surface kinetics and flow was solved in Python using the SciPy LSODA integrator^45^ and the BDF method, with relative and absolute tolerances of 1 × 10^–8^ and 1 × 10^–10^, respectively. More details and a discussion of model choices are provided in the Kinetics section of the SI, Table S4, and Figures S14 and S15.

## Supporting Information Available

XPS spectrum of Pd 3d of Pd and Au_50_Pd_50_, XPS spectrum of Pd 3d and Au 4f prior CO oxidation, SEM images of the samples, schematic of the experimental setup, raw data of light-off curves, absorption efficiency spectra and integrated absorption efficiency for different alloy compositions, CO_2_ ion current in the dark and under illumination, temperature difference in the dark and under illumination of the sample at different temperature steps, CO_2_ ion current in the dark and under illumination in the mass transport limited regime, mean diameter and height of samples after annealing, temperature increase upon illumination, transmission spectrum of empty glass pocket and intensity profile of lamp, explanation of first-principle calculations, side view of relaxed CO and O adsorbates for different surface configurations, top view of relaxed CO and O adsorbates for different surface configurations, binding energies of CO and O calculated by DFT, electronic and free energies calculated by DFT, explanation of considered CO oxidation kinetics, predicted conversion for different model systems, steady-state surface coverage as a function of temperature for different model systems (PDF)

## References

[ref1] Christopher P.; Xin H.; Linic S. Visible-Light-Enhanced Catalytic Oxidation Reactions on Plasmonic Silver Nanostructures. Nat. Chem. 2011, 3, 467–472. 10.1038/nchem.1032. 21602862

[ref2] Linic S.; Aslam U.; Boerigter C.; Morabito M. Photochemical Transformations on Plasmonic Metal Nanoparticles. Nat. Mater. 2015, 14, 567–576. 10.1038/nmat4281. 25990912

[ref3] Mukherjee S.; Libisch F.; Large N.; Neumann O.; Brown L. V.; Cheng J.; Lassiter J. B.; Carter E. A.; Nordlander P.; Halas N. J. Hot Electrons Do the Impossible: Plasmon-Induced Dissociation of H_2_ on Au. Nano Lett. 2013, 13, 240–247. 10.1021/nl303940z. 23194158

[ref4] Hou W.; Cronin S. B. A Review of Surface Plasmon Resonance-Enhanced Photocatalysis. Adv. Funct. Mater. 2013, 23, 1612–1619. 10.1002/adfm.201202148.

[ref5] Kale M. J.; Avanesian T.; Christopher P. Direct Photocatalysis by Plasmonic Nanostructures. ACS Catal. 2014, 4, 116–128. 10.1021/cs400993w.

[ref6] Smith J. G.; Faucheaux J. A.; Jain P. K. Plasmon Resonances for Solar Energy Harvesting: A Mechanistic Outlook. Nano Today 2015, 10, 67–80. 10.1016/j.nantod.2014.12.004.

[ref7] Tian Y.; Tatsuma T. Plasmon-Induced Photoelectrochemistry at Metal Nanoparticles Supported on Nanoporous TiO_2_. Chem. Commun. 2004, 1810–1811. 10.1039/b405061d. 15306895

[ref8] Nitzan A.; Brus L. E. Theoretical-Model for Enhanced Photochemistry on Rough Surfaces. J. Chem. Phys. 1981, 75, 2205–2214. 10.1063/1.442333.

[ref9] Brus L. Noble Metal Nanocrystals: Plasmon Electron Transfer Photochemistry and Single-Molecule Raman Spectroscopy. Acc. Chem. Res. 2008, 41, 1742–1749. 10.1021/ar800121r. 18783255

[ref10] Langhammer C.; Kasemo B.; Zoric I. Absorption and Scattering of Light by Pt, Pd, Ag, and Au Nanodisks: Absolute Cross Sections and Branching Ratios. J. Chem. Phys. 2007, 126, 194702 10.1063/1.2734550. 17523823

[ref11] Baffou G.; Quidant R. Nanoplasmonics for Chemistry. Chem. Soc. Rev. 2014, 43, 3898–3907. 10.1039/c3cs60364d. 24549257

[ref12] Seemala B.; Therrien A. J.; Lou M.; Li K.; Finzel J. P.; Qi J.; Nordlander P.; Christopher P. Plasmon-Mediated Catalytic O_2_ Dissociation on Ag Nanostructures: Hot Electrons or near Fields?. ACS Energy Letters 2019, 4, 1803–1809. 10.1021/acsenergylett.9b00990.

[ref13] Dubi Y.; Sivan Y. Hot″ Electrons in Metallic Nanostructures-Non-Thermal Carriers or Heating?. Light: Sci. Appl. 2019, 8, 89 10.1038/s41377-019-0199-x. 31645933 PMC6804576

[ref14] Dubi Y.; Un I. W.; Sivan Y. Thermal Effects – An Alternative Mechanism for Plasmon-Assisted Photocatalysis. Chemical Science 2020, 11, 5017–5027. 10.1039/C9SC06480J. 34122958 PMC8159236

[ref15] Sivan Y.; Baraban J.; Un I. W.; Dubi Y. Comment on ″Quantifying Hot Carrier and Thermal Contributions in Plasmonic Photocatalysis″. Science 2019, 364, eaaw9367 10.1126/science.aaw9367. 31048461

[ref16] Jain P. K. Comment on “Thermal Effects – An Alternative Mechanism for Plasmon-Assisted Photocatalysis” by Y. Dubi, I. W. Un and Y. Sivan, Chemical Science, 2020, 11, 5017. Chemical Science 2020, 11, 9022–9023. 10.1039/D0SC02914A. 34125118 PMC8163434

[ref17] Dubi Y.; Un I. W.; Sivan Y. Reply to the ‘Comment on “Thermal Effects–An Alternative Mechanism for Plasmon-Assisted Photocatalysis”’ by P. Jain, Chemical Science, 2020, 11, DOI: 10.1039/D0SC02914A. Chem. Sci. 2020, 11, 9024–9025. 10.1039/D0SC03335A. 34125112 PMC8163411

[ref18] Jain P. K. Taking the Heat Off of Plasmonic Chemistry. J. Phys. Chem. C 2019, 123, 24347–24351. 10.1021/acs.jpcc.9b08143.

[ref19] Sivan Y.; Baraban J. H.; Dubi Y. Experimental Practices Required to Isolate Thermal Effects in Plasmonic Photo-Catalysis: Lessons from Recent Experiments. OSA Continuum 2020, 3, 483–497. 10.1364/OSAC.376809.

[ref20] Baffou G.; Bordacchini I.; Baldi A.; Quidant R. Simple Experimental Procedures to Distinguish Photothermal from Hot-Carrier Processes in Plasmonics. Light: Sci. Appl. 2020, 9, 108 10.1038/s41377-020-00345-0. PMC7321931 32612818

[ref21] Bauer M.; Marienfeld A.; Aeschlimann M. Hot Electron Lifetimes in Metals Probed by Time-Resolved Two-Photon Photoemission. Prog. Surf. Sci. 2015, 90, 319–376. 10.1016/j.progsurf.2015.05.001.

[ref22] Zoric I.; Zäch M.; Kasemo B.; Langhammer C. Gold, Platinum, and Aluminum Nanodisk Plasmons: Material Independence, Subradiance, and Damping Mechanisms. ACS Nano 2011, 5, 2535–2546. 10.1021/nn102166t. 21438568

[ref23] Venezia A. M.; Liotta L. F.; Pantaleo G.; La Parola V.; Deganello G.; Beck A.; Koppany Z.; Frey K.; Horvath D.; Guczi L. Activity of SiO_2_ Supported Gold-Palladium Catalysts in CO Oxidation. Appl. Catal., A 2003, 251, 359–368. 10.1016/S0926-860X(03)00343-0.

[ref24] Gao F.; Goodman D. W. Pd–Au Bimetallic Catalysts: Understanding Alloy Effects from Planar Models and (Supported) Nanoparticles. Chem. Soc. Rev. 2012, 41, 8009–8020. 10.1039/c2cs35160a. 22824870

[ref25] Fredriksson H.; Alaverdyan Y.; Dmitriev A.; Langhammer C.; Sutherland D. S.; Zaech M.; Kasemo B. Hole-Mask Colloidal Lithography. Adv. Mater. 2007, 19, 4297–4302. 10.1002/adma.200700680.

[ref26] Nugroho F. A. A.; Iandolo B.; Wagner J. B.; Langhammer C. Bottom-Up Nanofabrication of Supported Noble Metal Alloy Nanoparticle Arrays for Plasmonics. ACS Nano 2016, 10, 2871–2879. 10.1021/acsnano.5b08057. 26828308

[ref27] Fredriksson H. O. A.; Larsson Langhammer E. M.; Niemantsverdriet J. W. Reduction of Cu-Promoted Fe Model Catalysts Studied by *in Situ* Indirect Nanoplasmonic Sensing and X-Ray Photoelectron Spectroscopy. J. Phys. Chem. C 2015, 119, 4085–4094. 10.1021/jp511596s.

[ref28] Bu Y.; Niemantsverdriet J. W. H.; Fredriksson H. O. A. Cu Model Catalyst Dynamics and CO Oxidation Kinetics Studied by Simultaneous *in Situ* UV–Vis and Mass Spectroscopy. ACS Catal. 2016, 6, 2867–2876. 10.1021/acscatal.5b02861.

[ref29] Falsig H.; Hvolbaek B.; Kristensen I. S.; Jiang T.; Bligaard T.; Christensen C. H.; Norskov J. K. Trends in the Catalytic CO Oxidation Activity of Nanoparticles. Angew. Chem., Int. Ed. 2008, 47, 4835–4839. 10.1002/anie.200801479. 18496809

[ref30] Zhang J.; Jin H.; Sullivan M. B.; Lim F. C.; Wu P. Study of Pd-Au Bimetallic Catalysts for CO Oxidation Reaction by DFT Calculations. Phys. Chem. Chem. Phys. 2009, 11, 1441–1446. 10.1039/b814647k. 19224045

[ref31] Rahm J. M.; Tiburski C.; Rossi T. P.; Nugroho F. A. A.; Nilsson S.; Langhammer C.; Erhart P. A Library of Late Transition Metal Alloy Dielectric Functions for Nanophotonic Applications. Adv. Funct. Mater. 2020, 30, 2002122 10.1002/adfm.202002122.

[ref32] Langhammer C.; Schwind M.; Kasemo B.; Zorić I. Localized Surface Plasmon Resonances in Aluminum Nanodisks. Nano Lett. 2008, 8, 1461–1471. 10.1021/nl080453i. 18393471

[ref33] Langhammer C.; Yuan Z.; Zoric I.; Kasemo B. Plasmonic Properties of Supported Pt and Pd Nanostructures. Nano Lett. 2006, 6, 833–838. 10.1021/nl060219x. 16608293

[ref34] Sytwu K.; Vadai M.; Dionne J. A. Bimetallic Nanostructures: Combining Plasmonic and Catalytic Metals for Photocatalysis. Advances in Physics: X 2019, 4, 1619480 10.1080/23746149.2019.1619480.

[ref35] Zhdanov V. P.; Zorić I.; Kasemo B. Plasmonics: Heat Transfer between Metal Nanoparticles and Supporting Nanolayers. Phys. E 2012, 46, 113–118. 10.1016/j.physe.2012.09.004.

[ref36] Fredriksson H.; Alaverdyan Y.; Dmitriev A.; Langhammer C.; Sutherland D. S.; Zaech M.; Kasemo B. Hole-Mask Colloidal Lithography. Adv. Mater. 2007, 19, 4297–4302. 10.1002/adma.200700680.

[ref37] Nugroho F. A. A.; Iandolo B.; Wagner J. B.; Langhammer C. Bottom-Up Nanofabrication of Supported Noble Metal Alloy Nanoparticle Arrays for Plasmonics. ACS Nano 2016, 10, 2871–2879. 10.1021/acsnano.5b08057. 26828308

[ref38] Grant A. W.; Hu Q. H.; Kasemo B. Transmission Electron Microscopy ’Windows’ for Nanofabricated Structures. Nanotechnology 2004, 15, 1175–1181. 10.1088/0957-4484/15/9/012.

[ref39] Palik E. D. Handbook of Optical Constants of Solids; Academic press: Orlando, 1998; Vol. 3.

[ref40] Greczynski G.; Hultman L. X-Ray Photoelectron Spectroscopy: Towards Reliable Binding Energy Referencing. Prog. Mater. Sci. 2020, 107, 100591 10.1016/j.pmatsci.2019.100591.

[ref41] Kresse G.; Hafner J. *ab initio* Molecular Dynamics for Open-Shell Transition Metals. Phys. Rev. B: Condens. Matter Mater. Phys. 1993, 48, 13115–13118. 10.1103/PhysRevB.48.13115. 10007687

[ref42] Kresse G.; Furthmuller J. Efficiency of *ab-Initio* Total Energy Calculations for Metals and Semiconductors Using a Plane-Wave Basis Set. Comput. Mater. Sci. 1996, 6, 15–50. 10.1016/0927-0256(96)00008-0. 9984901

[ref43] Hammer B.; Hansen L. B.; Norskov J. K. Improved Adsorption Energetics within Density-Functional Theory Using Revised Perdew-Burke-Ernzerhof Functionals. Phys. Rev. B: Condens. Matter Mater. Phys. 1999, 59, 7413–7421. 10.1103/PhysRevB.59.7413.

[ref44] Blochl P. E. Projector Augmented-Wave Method. Phys. Rev. B: Condens. Matter Mater. Phys. 1994, 50, 17953–17979. 10.1103/PhysRevB.50.17953. 9976227

[ref45] Petzold L. Automatic Selection of Methods for Solving Stiff and Nonstiff Systems of Ordinary Differential-Equations. Siam Journal on Scientific and Statistical Computing 1983, 4, 136–148. 10.1137/0904010.

