# Supplementary material for: Light-Off in Plasmon-Mediated Photocatalysis
Source: ACS Nano. 2021 Jun 22;15(7):11535–42. doi: 10.1021/acsnano.1c01537 (PMC8320230; doi:10.1021/acsnano.1c01537)
Supplement: Supplementary file 1 — nn1c01537_si_001.pdf [file nn1c01537_si_001.pdf]

Supporting Information
for
Light-Off in Plasmon-Mediated Photocatalysis

Christopher Tiburski[1], Astrid Boje[1], Sara Nilsson[1], Zafer Say[1], Joachim Fritzsche[1], Henrik Ström[2], Anders Hellman[1], Christoph Langhammer [1,]*

[1] Department of Physics, Chalmers University of Technology, 412 96 Göteborg, Sweden

[2] Department of Mechanics and Maritime Sciences, Chalmers University of Technology, 412 96 Göteborg, Sweden.

*Correspondence to: clangham@chalmers.se

Supplementary Figures

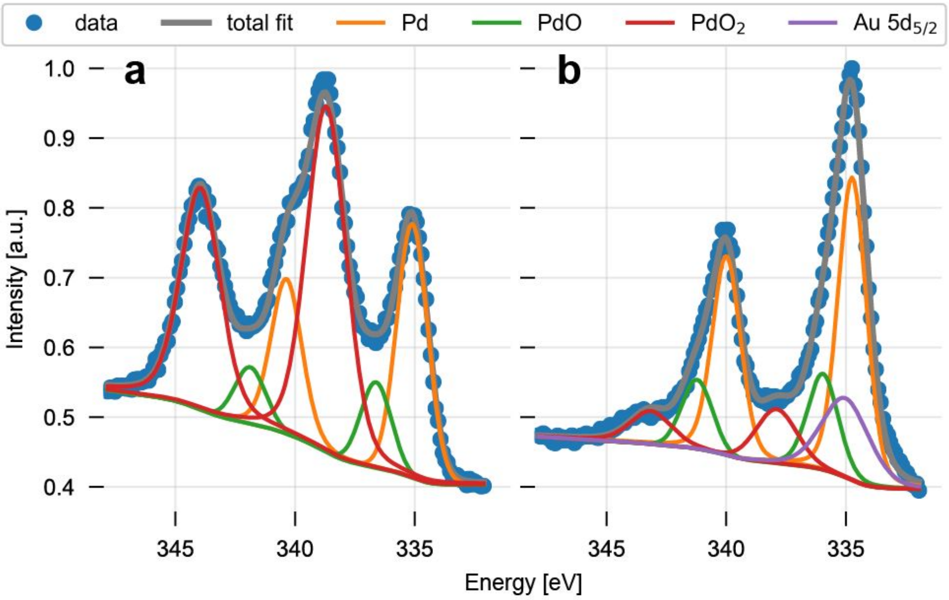

Figure S1. *XPS spectrum of the Pd3d peak after multiple runs of CO-oxidation measurements on a) Pd and b) $Au_{50}Pd_{50}$. Both samples exhibit different amounts of palladium oxides on the surface, in good agreement with the literature.*[1,2]

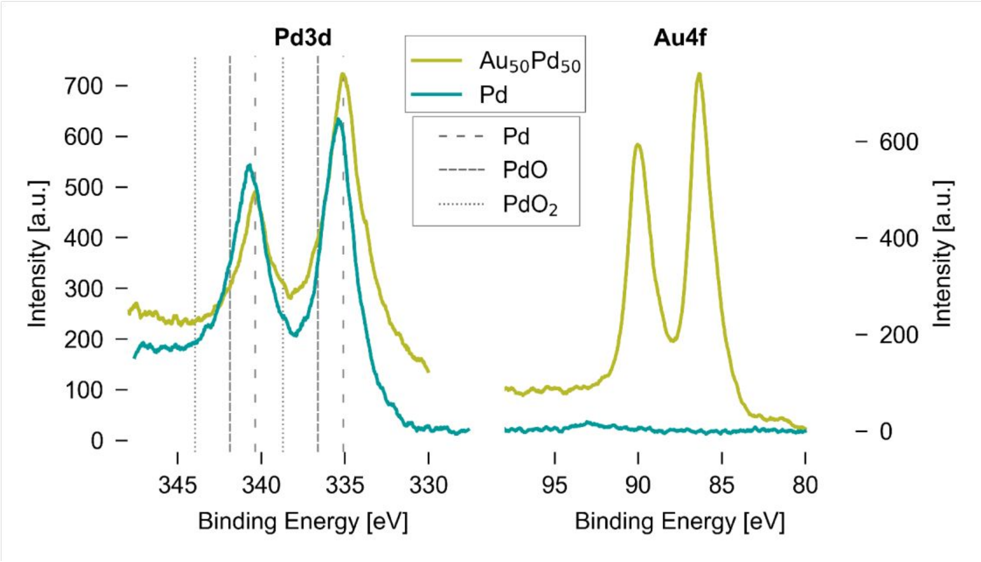

Figure S2 *XPS spectra of a neat Pd and an $Au_{50}Pd_{50}$ alloy nanoparticle array for the Pd3d (left) and Au4f (right) peak regions before CO oxidation measurements. The spectra reveal mostly metallic constituents.*

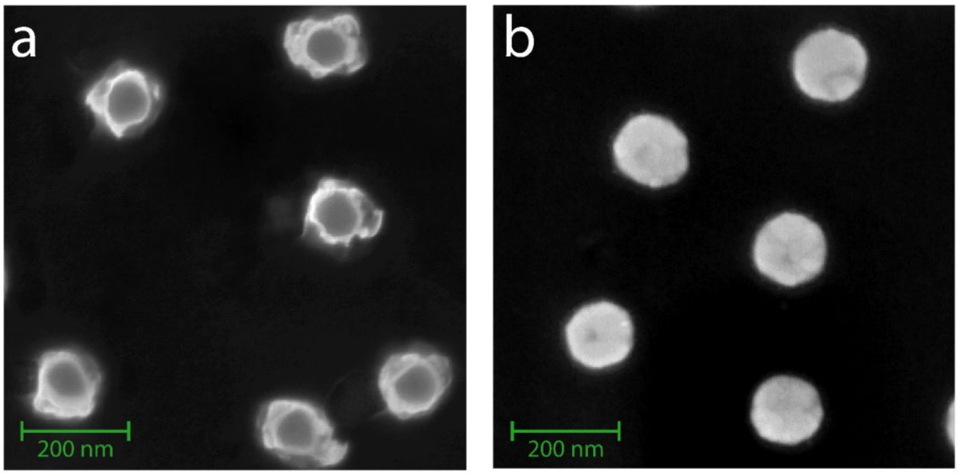

Figure S3. *SEM performed on the neat Pd (a) and $Au_{50}Pd_{50}$ (b) samples after the CO oxidation experiments. The obtained images underpin the XPS results since the palladium particles show clear signs of oxidation, whereas the gold-palladium alloy sample does not show equally strong structural signs of oxidation.*

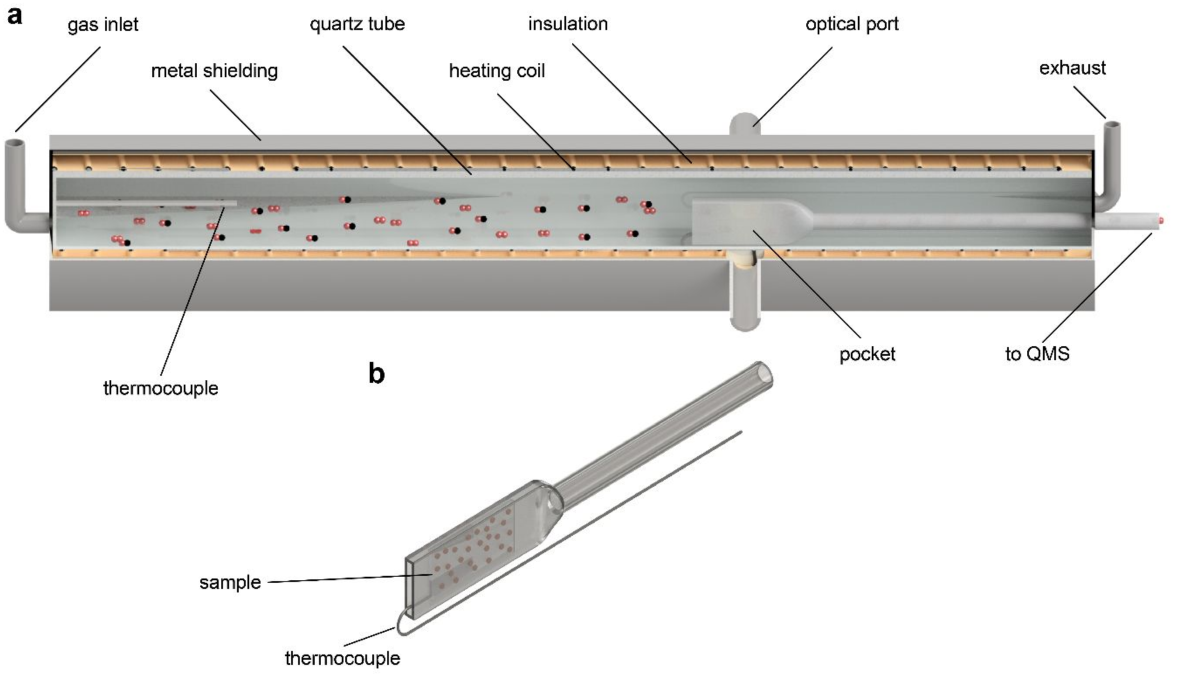

Figure S4. *a) Schematic of the quartz tube plug-flow reactor with its external resistive heating coil and metal shielding. The gas inlet is connected to an array of mass-flow-controllers. The pocket can be illuminated via an optical port. Reaction products are analyzed by a quadrupole-mass-spectrometer (QMS) connected to the outlet of the pocket. Thermocouples monitor gas temperature upstream of the pocked and sample temperature inside the pocket. The optical ports were used to illuminate the sample with a mercury xenon arc light source (**Fig. S12**). b) Schematic of the pocked, in which the nanofabricated sample is mounted. A spring-loaded thermocouple ensures good thermal contact with the side of the sample and keeps track of its temperature.*

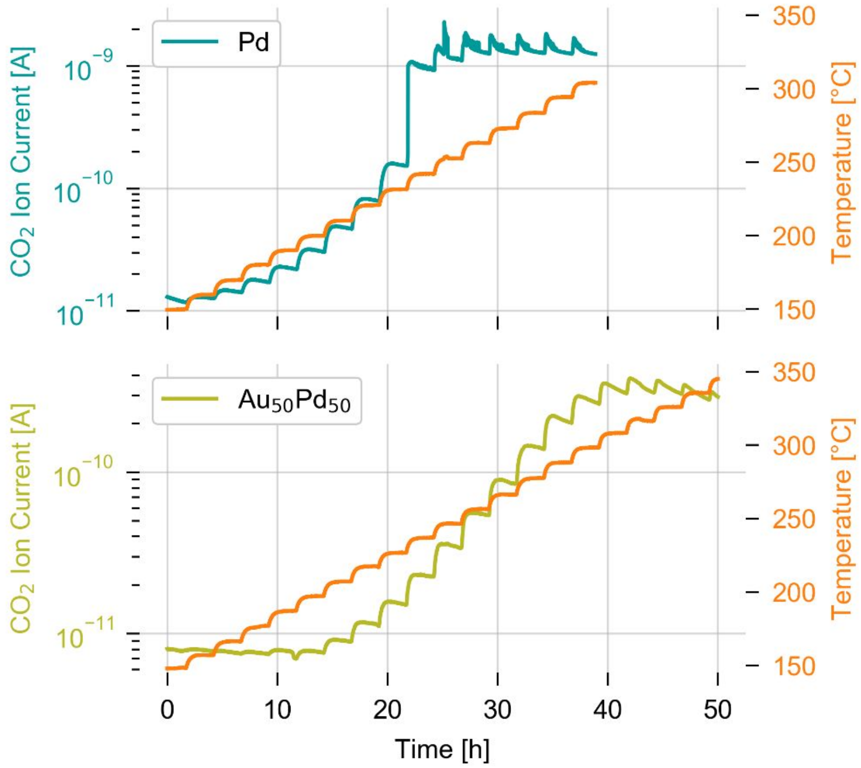

Figure S5. *Raw data for light-off curve determination in the dark. Blue and yellow curves depict the CO_2 ion current for the Pd and $Au_{50}Pd_{50}$ sample, respectively. Orange curves depict the sample temperature measured by the thermocouple mounted on the back side of the sample. The temperature was increased in steps and for the CO_2 ion current signal the last 25 min of each step were averaged. The spikes that appear in the ion current of the Pd sample in the plateau region are likely kinetic oscillations related to the catalyst surface oscillating between a reduced and oxidized state.[3] The observed slight catalyst deactivation after each temperature step we tentatively attribute to the formation of less active Pd-oxide species.[1,4]*

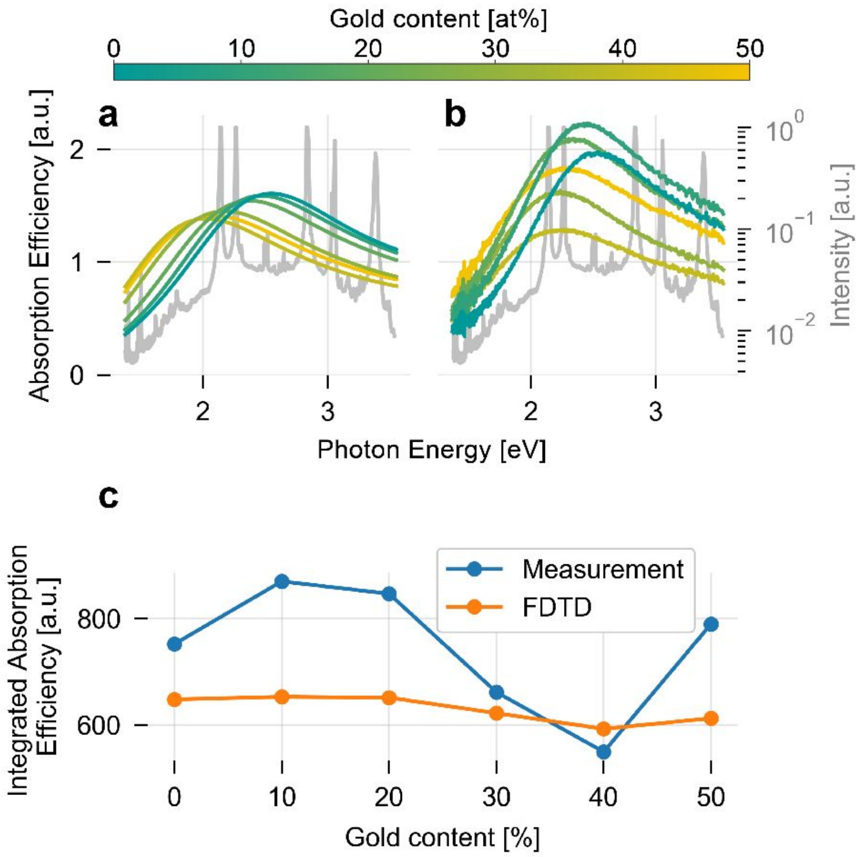

Figure S6. *Unscaled Light absorption efficiency in Pd and Au_xPd_{100-x} alloy nanodisks for x = 10, 20, 30, 40, 50. Same data as in Fig. 3 in the main text but without scaling the experimentally measured absorption efficiency spectra to the FDTD-simulated spectra.*

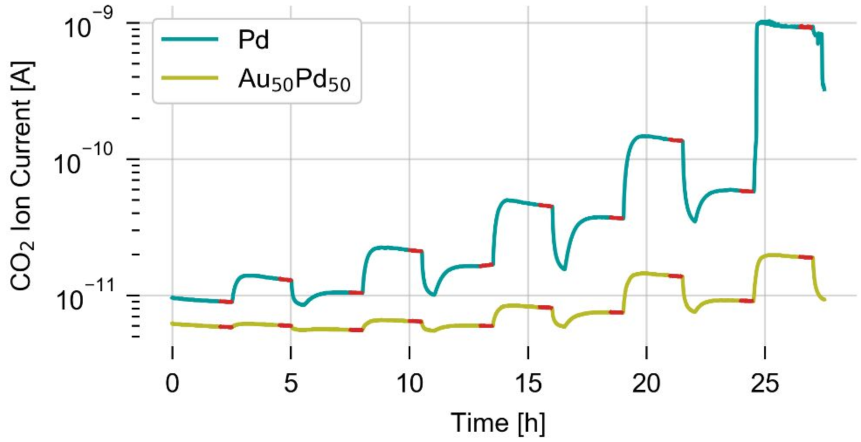

Figure S7. *The CO_2 ion current in the dark and upon illumination for neat Pd (blue) and $Au_{50}Pd_{50}$ (yellow) measured in a constant flow of CO and O_2 in Ar carrier gas with α^{CO} = 0.2 and at five different reactor temperatures ranging from ~150°C to ~220°C. The signal from the red-marked sections is averaged to calculate CO conversion at each step.*

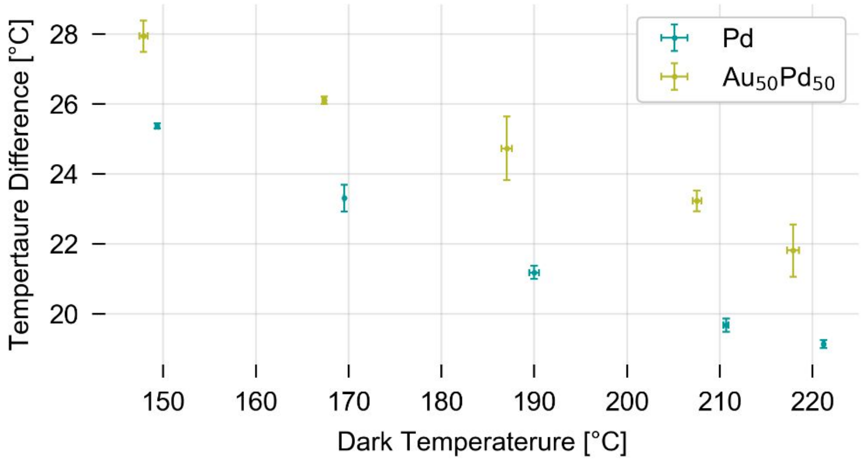

Figure S8. *Temperature difference of the catalyst measured with a thermocouple on the side of the sample during CO-oxidation in the dark and under illumination at five different reactor temperatures. The small difference between Pd and $Au_{50}Pd_{50}$ we attribute to small differences in how the lamp and thermocouple were mounted in the two experiments.*

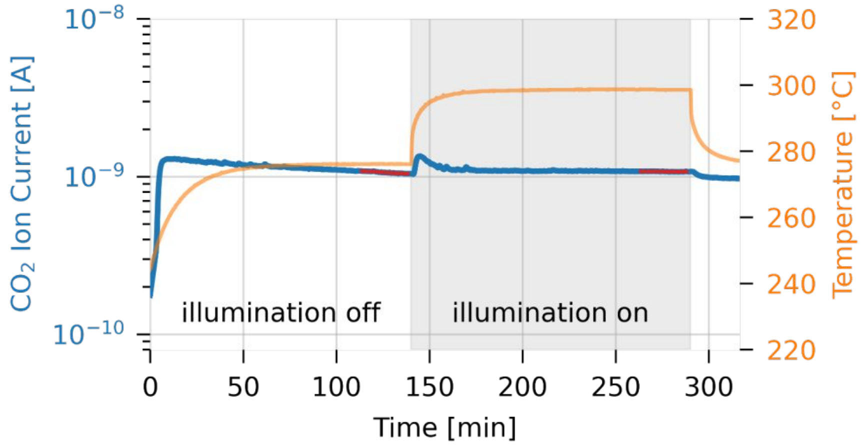

Figure S9. *The CO_2 ion current in the dark and upon illumination for neat Pd measured in the fully mass transport limited regime in a constant flow of CO and O_2 in Ar carrier gas with α^{CO} = 0.2 and at a reactor temperature of ~275°C. The signal from the red-marked sections is averaged to calculate CO conversion.*

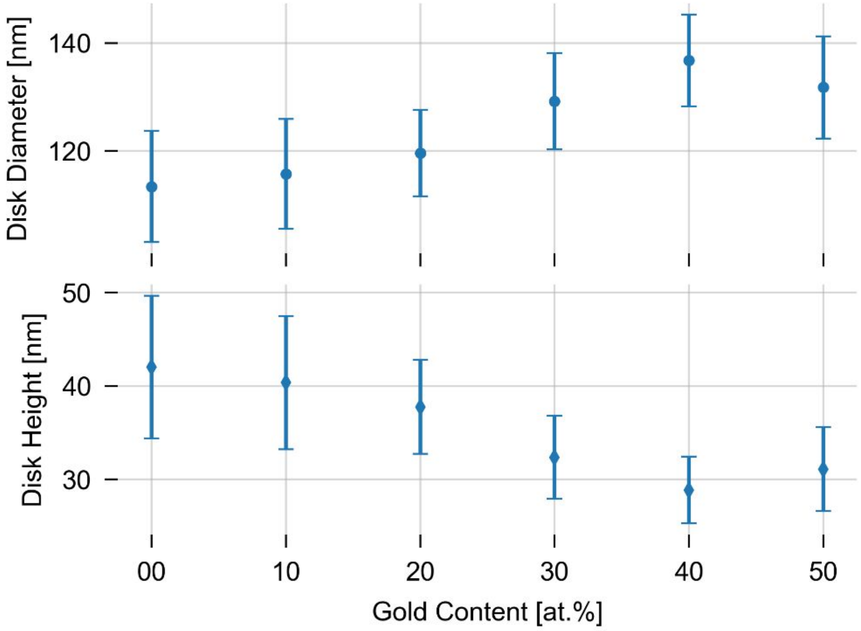

Figure S10. *The mean diameter of the nanodisks for alloys with different composition was derived from SEM images by analyzing at least 350 particles from vastly different sample positions. The disk height was then calculated by assuming volume conservation from the unannealed to the annealed particle.*

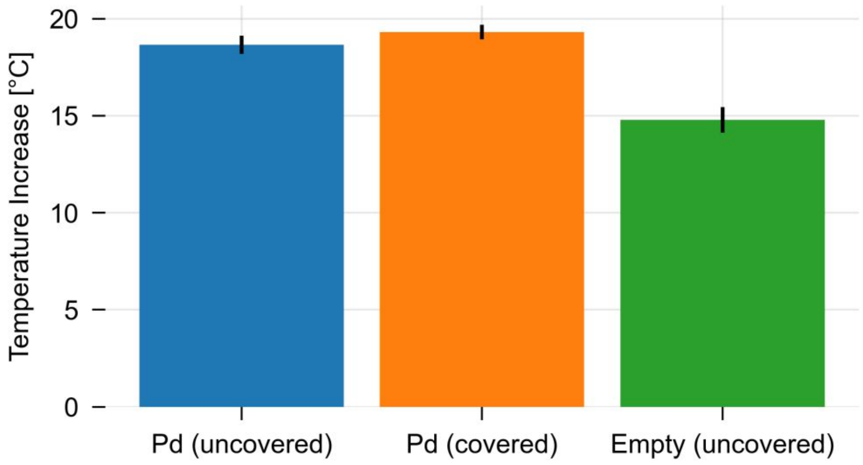

Figure S11. *The measured temperature increase upon illumination of 5W/cm² at a set reactor temperature of ~162°C without a sample, a Pd sample, and a Pd sample with the thermocouple shielded from direct illumination in the reactor. The latter two measurements show the influence of direct illumination on the thermocouple with a Pd nanoparticle sample in the reactor, which is on the order of 1°C or less. Comparing the measured temperature increase for the empty reactor with the case of a Pd nanoparticle sample in the reactor reveals a difference of several degrees, which corroborates a heating effect by the particles and efficient heat dissipation from the particles to the substrate.*

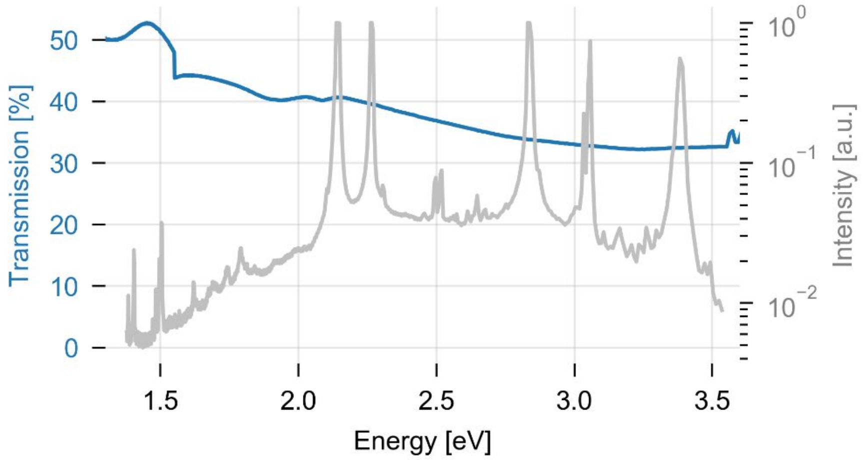

Figure S12. *Optical transmission measured through the reactor with mounted pocket, plotted together with the emission spectrum of the mercury xenon arc light source used. The spectrum is measured through the setup (fig. S4) and excludes therefore IR-radiation, which is absorbed in the IR filter and UV-radiation, which is blocked by the quartz and glass walls of the reactor tube and pocket, respectively.*

Microkinetic model

A first principles informed microkinetic model was constructed to investigate the different convergence profiles obtained for the pure palladium and palladium-gold alloy catalysts. Here we outline the first principles calculations and present the kinetic model.

First principles calculations

Four systems were constructed to model the palladium and palladium-gold systems. Pd(111) was used as the model for the pure Pd catalyst. For the alloy system, a 50:50 mix of Pd and Au was configured in three different surface models to assess the impact of surface configuration on adsorbate interactions: a mixed top layer, a Pd top layer, and an Au top layer. In the models with segregated top layers, the second layer contains the other atom type, and all other layers are evenly mixed.

The relaxed geometries and electronic energies of the clean surfaces and the surfaces with CO and O adsorbates were calculated in 2×2 unit cells, using density functional theory (DFT) in VASP[5,6] with the RPBE functional[7] and the projector augmented wave (PAW) method.[8] The plane wave basis set energy cutoff was 450 eV. A $8 \times 8 \times 1$ k-point mesh was used to sample the Brillouin zone. CO and O gas molecules were relaxed in a $10 \times 10 \times 10$ Å^3 box. The relaxed structures are shown in Table S1 and Table S2.

Activation energies, E_a, for formation of CO_2 on each surface were computed using the scaling relation developed to predict the transition state energies, E_TS, for CO oxidation by Falsig *et al.*[9], *i.e.*,

$$E_\mathrm{TS} = 0.70(E_\mathrm{O} + E_\mathrm{CO}) + 0.02$$

$$E_\mathrm{a} = \max\left(E_\mathrm{TS} - (E_\mathrm{O} + E_\mathrm{CO}), 0\right)$$

As seen in Table S3, the model with Pd as a top layer behaves similarly to the pure Pd model, while the mixed alloy has a slightly lower barrier, and the Au top layer was found to have no barrier after taking the maximum shown above. These findings agree with previous DFT results.[10]

The free energies of each state were computed by adding entropic contributions from translational, rotational, and vibrational degrees of freedom to the activation energy (see Table S3), with adsorbate energies calculated in the harmonic limit and gases using the ideal gas

approximation.[11] For the empty surfaces, the Au top layer was found to be the most favorable configuration, followed by the mixed top layer, with the Pd top layer least favored (**Figure**).

***Table S1**: Side view of relaxed CO and O adsorbate structures with surface extended periodically beyond the unit cell for the purpose of visualization only.*

	CO	O
Pd(111)	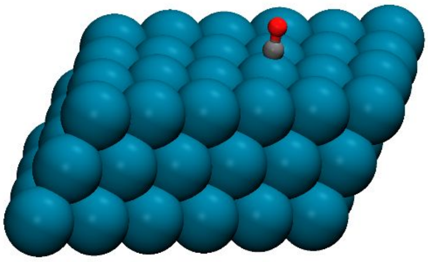	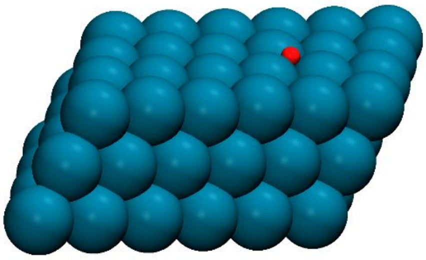
PdAu alloy, mixed top layer	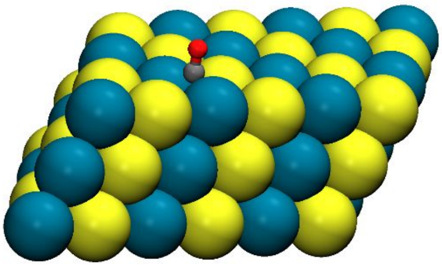	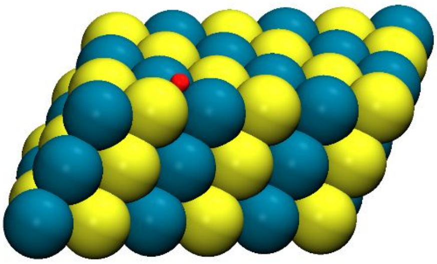
PdAu alloy, Pd top layer	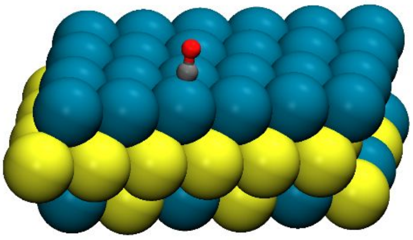	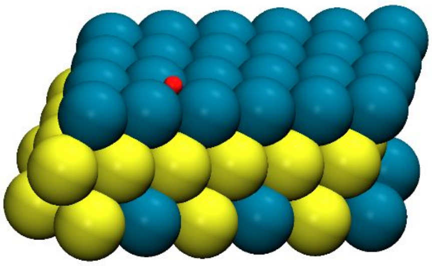
PdAu alloy, Au top layer	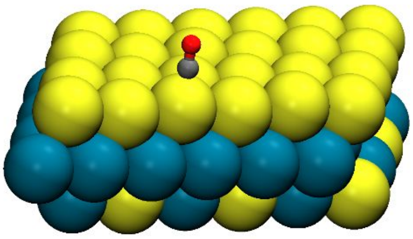	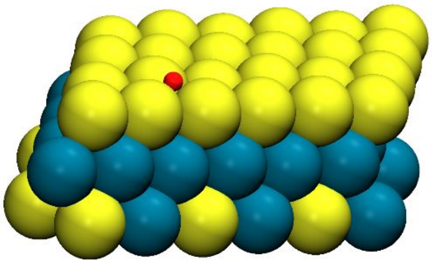

Table S2: *Top view of relaxed CO and O adsorbate structures with surface extended periodically beyond the unit cell for the purpose of visualization only.*

	CO	O
Pd(111)	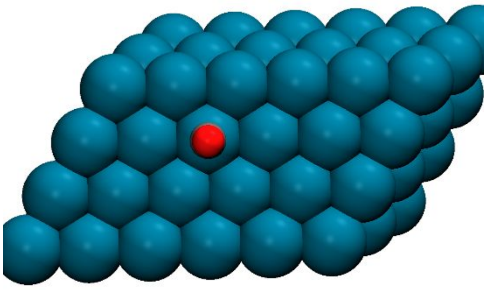	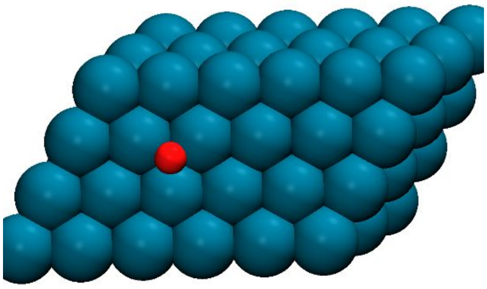
PdAu alloy, mixed top layer	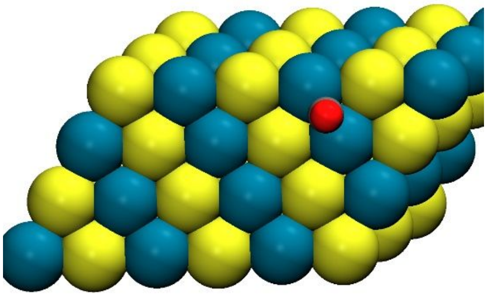	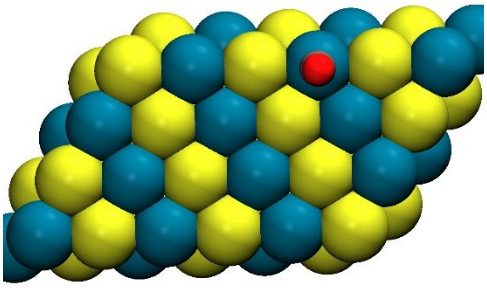
PdAu alloy, Pd top layer	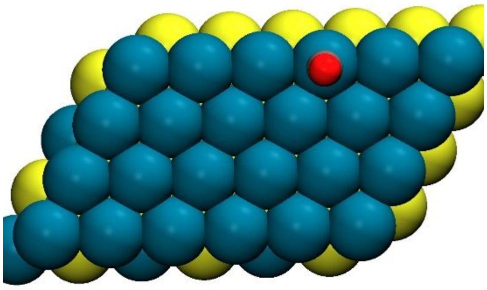	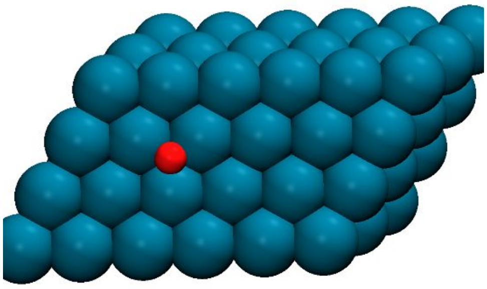
PdAu alloy, Au top layer	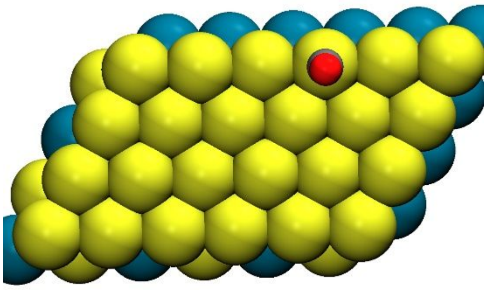	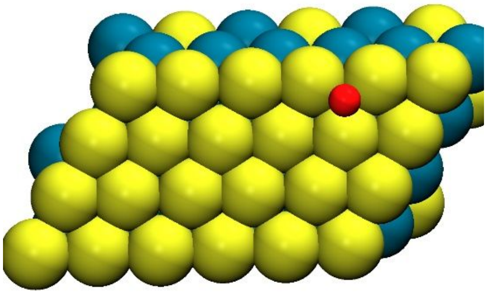

Table S3: *Binding energies of CO and O calculated using DFT, relative to the clean surface and respective gas molecule, and transition state (TS) energy and activation energy (* $\boldsymbol{E_a}$ *) calculated using a scaling relation.*

	E_{CO} (eV)	E_{O} (eV)	E_{TS} (eV)	E_{a} (eV)
Pd(111)	-1.18	-1.14	-1.61	0.717
PdAu alloy, mixed top layer	-1.34	-0.49	-1.26	0.568
PdAu alloy, Pd top layer	-1.14	-1.15	-1.58	0.706
PdAu alloy, Au top layer	-0.0936	0.34	0.196	0.0

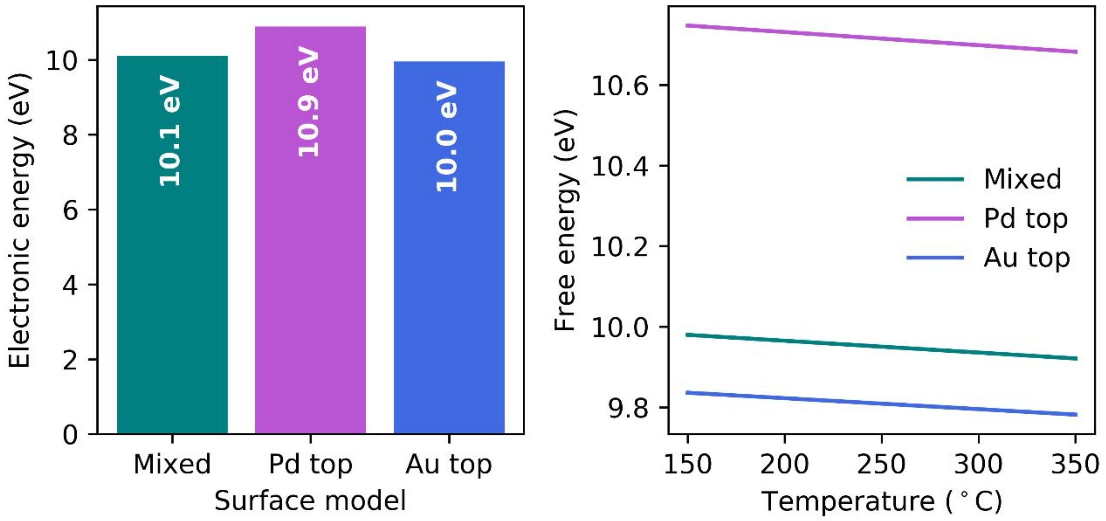

Figure S13: *Electronic (left) and free (right) energies calculated with DFT and the rigid-rotor, harmonic-oscillator approximation for clean alloy surfaces with mixed, Pd and Au top layers relative to the Pd(111) surface.*

Kinetics

The following elementary steps were considered, describing adsorption/desorption of CO, dissociative adsorption/desorption of O_2 and irreversible reaction of adsorbed CO and O to form gaseous CO_2 respectively:

$$\mathrm{CO_{(g)}} + * \leftrightarrow \mathrm{CO}^*$$
$$\mathrm{O_{2(g)}} + 2* \leftrightarrow 2\mathrm{O}^*$$
$$\mathrm{CO}^* + \mathrm{O}^* \rightarrow 2* + \mathrm{CO_{2(g)}}$$

Here, * refers to a free site and the type is either Pd or Au depending on the system. The adsorption rate constants were taken from collision theory,

$$k_{\mathrm{ads}} = \frac{A}{\sqrt{2\pi m k_{\mathrm{B}} T}},$$

where A is the site area (estimated from the van der Waals radius of the atom), m is the mass of the adsorbing molecule, k_{B} is the Boltzmann constant and T is the temperature. The desorption processes were assumed to be in thermal equilibrium,

$$K_{\mathrm{eq}} = \frac{k_{\mathrm{ads}}}{k_{\mathrm{des}}} = \exp\left(-\frac{G_{\mathrm{rxn}}}{RT}\right),$$

where G_{rxn} is the binding energy (see Table S3) and R is the gas constant. The oxidation reaction was assumed to have Langmuir-Hinshelwood kinetics, with Arrhenius rate constant,

$$k_{\mathrm{LH}} = \left(\frac{k_{\mathrm{B}}T}{h}\right)\exp\left(-\frac{G_{\mathrm{a}}}{RT}\right).$$

Here, h is the Planck constant and G_{a} is the activation free energy.

For each model system, the surface kinetics were described by the following pair of equations,

$$\frac{d\theta_{\mathrm{CO}}}{dt} = k_{\mathrm{ads}}^{\mathrm{CO}} P_{\mathrm{CO}} \theta_* - k_{\mathrm{des}}^{\mathrm{CO}} \theta_{\mathrm{CO}} - k_{\mathrm{LH}} \theta_{\mathrm{CO}} \theta_{\mathrm{O}}$$
$$\frac{d\theta_{\mathrm{O}}}{dt} = 2k_{\mathrm{ads}}^{\mathrm{O_2}} P_{\mathrm{O_2}} \theta_*^2 - 2k_{\mathrm{des}}^{\mathrm{O_2}} \theta_{\mathrm{O}}^2 - k_{\mathrm{LH}} \theta_{\mathrm{CO}} \theta_{\mathrm{O}},$$

where P_{CO} and $P_{\mathrm{O_2}}$are partial pressures and θ is the fractional surface coverage. The unoccupied site fraction was calculated as $\theta_* = 1 - \theta_{\mathrm{CO}} - \theta_{\mathrm{O}}$. The system kinetics were described by three equations:

$$\frac{dP_{\text{CO}}}{dt} = \frac{1}{\tau}\left(P_{\text{CO}}^{\text{in}} - P_{\text{CO}}\right) + \sigma\left(k_{\text{des}}^{\text{CO}}\theta_{\text{CO}} - k_{\text{ads}}^{\text{CO}}P_{\text{CO}}\theta_{*}\right)$$

$$\frac{dP_{\text{O}_2}}{dt} = \frac{1}{\tau}\left(P_{\text{O}_2}^{\text{in}} - P_{\text{O}_2}\right) + \sigma\left(k_{\text{des}}^{\text{O}_2}\theta_{\text{O}}^2 - k_{\text{ads}}^{\text{O}_2}P_{\text{O}_2}\theta_{*}^2\right)$$

$$\frac{dP_{\text{CO}_2}}{dt} = \frac{1}{\tau}\left(P_{\text{CO}_2}^{\text{in}} - P_{\text{CO}_2}\right) + \sigma\left(k_{\text{LH}}\theta_{\text{CO}}\theta_{\text{O}}\right).$$

That is, the reactor was modelled as a continuously stirred tank reactor (CSTR). The differential equations were solved in Python using the SciPy LSODA integrator[12] and the BDF method. Relative and absolute tolerances of 1×10^{-8} and 1×10^{-10} were specified.

The residence time, τ, was chosen to match the dimensions and flow rate of the experimental reactor and the inlet concentrations, $P_{\text{CO}}^{\text{in}}$ and $P_{\text{O}_2}^{\text{in}}$, were set to match the experimental conditions (

Table S4).

***Table S4**: Simulated reactor conditions.*

Residence time, τ (s)	4.5
CO inlet pressure, $P_{\text{CO}}^{\text{in}}$ (bar)	0.02
O_2 inlet pressure, $P_{\text{O}_2}^{\text{in}}$ (bar)	0.08
Total system pressure (bar)	1.0

The scaling constant, σ, which describes the conversion of the site-based rate to a pressure-based rate accounting for the number of catalyst sites in the reactor volume, was chosen to roughly predict the correct maximum conversion observed experimentally for the Pd and PdAu systems. This parameter was necessarily chosen separately for both systems, as it is challenging to obtain an accurate estimate for the number of exposed surface sites in each case.

With a suitable choice of the number of active sites, the Pd(111) model was found to provide good agreement with the experimental profile and light-off behavior of the pure palladium system (**Figure S14**, teal line). The reaction light-off occurs as the surface coverage is shifting from CO-dominated to mixed CO and O coverage (**Figure S15**). For the alloy model with a Pd top layer, the profiles obtained were very similar to the pure Pd case (**Figure S14** and **Figure S15**, blue lines), reflecting the similar energetics for this system. For the alloy model with the

Au top layer, it was possible to match approximately the smoother, lower experimental conversion profile by suitable choice of number of active sites although the barrierless oxidation reaction in this case over-predicted the conversion at low temperatures (**Figure S14**, salmon line). The low binding affinities, especially of oxygen, yield a virtually empty surface at all temperatures (**Figure S15**), suggesting significant mass transfer limitations exist. Finally, for the perfectly mixed alloy model, the conversion profile (**Figure S14**, purple line) was observed to have a similar shape to the Au top layer model owing to the reduced kinetic barrier; however, the higher binding affinities for both reactants resulted in higher surface concentrations (**Figure S15**) and correspondingly higher conversion. To match the low experimental conversion on the alloy with the perfectly mixed model, the number of active sites would need to be significantly reduced, resulting in an unexpectedly low site density compared to the other systems. Thus, it is postulated that the (most energetically favorable) Au top layer model best describes the alloy surface although a better model for the surface might be obtained by mixing the configurations investigated here.

In general, the models tested here indicate that inclusion of gold in the catalyst surface would tend to reduce both the adsorbate interaction with the surface and the reaction barrier, leading to low surface coverage – thus low conversion – but with the lower barrier reducing the kinetic limitations that are responsible for the light-off profile in the Pd dominated systems.

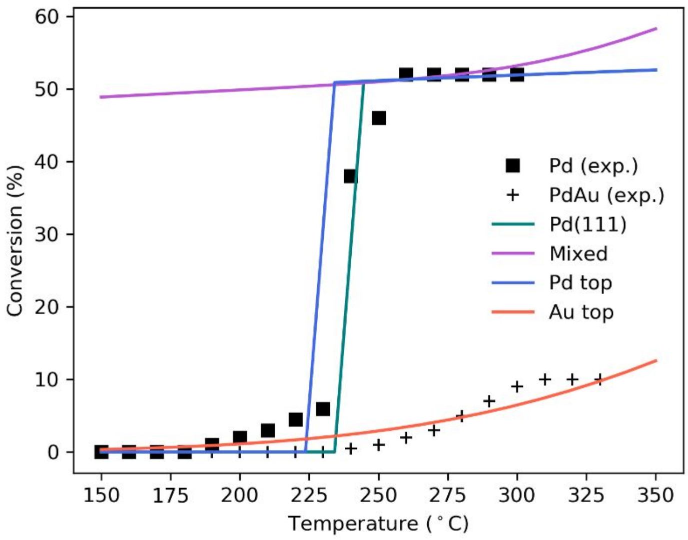

Figure S14: *Predicted conversion profiles for each model system with catalyst surface area scaled to achieve a desired maximum conversion.*

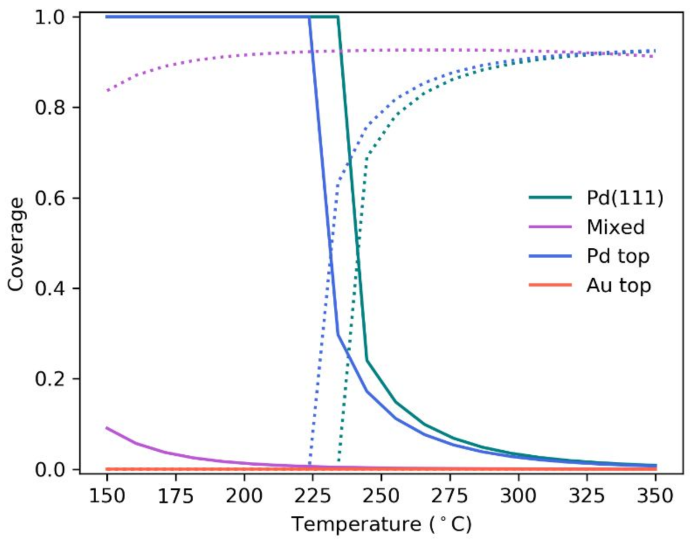

***Figure S15**: Steady-state surface coverage as a function of temperature for each model system. Solid lines show coverage of CO and dotted lines show coverage of O.*

References

1 Toyoshima, R., Yoshida, M., Monya, Y., Kousa, Y., Suzuki, K., Abe, H., Mun, B. S., Mase, K., Amemiya, K. & Kondoh, H. *In Situ* Ambient Pressure XPS Study of CO Oxidation Reaction on Pd(111) Surfaces. *The Journal of Physical Chemistry C*, 18691-18697, doi:10.1021/jp301636u (**2012**).

2 Venezia, A. M., Liotta, L. F., Pantaleo, G., La Parola, V., Deganello, G., Beck, A., Koppany, Z., Frey, K., Horvath, D. & Guczi, L. Activity of SiO_2 Supported Gold-Palladium Catalysts in CO Oxidation. *Applied Catalysis A-General*, 359-368, doi:10.1016/S0926-860x(03)00343-0 (**2003**).

3 Imbihl, R. & Ertl, G. Oscillatory Kinetics in Heterogeneous Catalysis. *Chemical Reviews*, 697-733 (**1995**).

4 Peterson, E. J., DeLaRiva, A. T., Lin, S., Johnson, R. S., Guo, H., Miller, J. T., Hun Kwak, J., Peden, C. H., Kiefer, B., Allard, L. F., Ribeiro, F. H. & Datye, A. K. Low-Temperature Carbon Monoxide Oxidation Catalysed by Regenerable Atomically Dispersed Palladium on Alumina. *Nat Commun*, 4885, doi:10.1038/ncomms5885 (**2014**).

5 Kresse, G. & Hafner, J. *Ab Initio* Molecular Dynamics for Open-Shell Transition Metals. *Physical Review B*, 13115-13118, doi:10.1103/physrevb.48.13115 (**1993**).

6 Kresse, G. & Furthmuller, J. Efficiency of *ab-initio* Total Energy Calculations for Metals and Semiconductors Using a Plane-Wave Basis Set. *Computational Materials Science*, 15-50, doi:Doi 10.1016/0927-0256(96)00008-0 (**1996**).

7 Hammer, B., Hansen, L. B. & Norskov, J. K. Improved Adsorption Energetics within Density-Functional Theory Using Revised Perdew-Burke-Ernzerhof Functionals. *Physical Review B*, 7413-7421, doi:DOI 10.1103/PhysRevB.59.7413 (**1999**).

8 Blochl, P. E. Projector Augmented-Wave Method. *Physical Review B*, 17953-17979, doi:10.1103/physrevb.50.17953 (**1994**).

9 Falsig, H., Hvolbaek, B., Kristensen, I. S., Jiang, T., Bligaard, T., Christensen, C. H. & Norskov, J. K. Trends in the Catalytic CO Oxidation Activity of Nanoparticles. *Angewandte Chemie*, 4835-4839, doi:10.1002/anie.200801479 (**2008**).

10 Zhang, J., Jin, H., Sullivan, M. B., Lim, F. C. & Wu, P. Study of Pd-Au Bimetallic Catalysts for CO Oxidation Reaction by DFT Calculations. *Physical Chemistry Chemical Physics*, 1441-1446, doi:10.1039/b814647k (**2009**).

11 Jensen, F. *Introduction to Computational Chemistry*. (John wiley & sons, New York, **2017**).

12 Petzold, L. Automatic Selection of Methods for Solving Stiff and Nonstiff Systems of Ordinary Differential-Equations. *Siam Journal on Scientific and Statistical Computing*, 136-148, doi:Doi 10.1137/0904010 (**1983**).
